# A Developmental Systems Perspective on Epistasis: Computational Exploration of Mutational Interactions in Model Developmental Regulatory Networks

**DOI:** 10.1371/journal.pone.0006823

**Published:** 2009-09-07

**Authors:** Jayson Gutiérrez

**Affiliations:** Grupo de Física y Astrofísica Computacional (FACom), Instituto de Física, Universidad de Antioquia, Medellín, Colombia; Centre for Genomic Regulation, Spain

## Abstract

The way in which the information contained in genotypes is translated into complex phenotypic traits (i.e. embryonic expression patterns) depends on its decoding by a multilayered hierarchy of biomolecular systems (regulatory networks). Each layer of this hierarchy displays its own regulatory schemes (i.e. operational rules such as +/− feedback) and associated control parameters, resulting in characteristic variational constraints. This process can be conceptualized as a mapping issue, and in the context of highly-dimensional genotype-phenotype mappings (GPMs) epistatic events have been shown to be ubiquitous, manifested in non-linear correspondences between changes in the genotype and their phenotypic effects. In this study I concentrate on epistatic phenomena pervading levels of biological organization above the genetic material, more specifically the realm of molecular networks. At this level, systems approaches to studying GPMs are specially suitable to shed light on the mechanistic basis of epistatic phenomena. To this aim, I constructed and analyzed ensembles of highly-modular (fully interconnected) networks with distinctive topologies, each displaying dynamic behaviors that were categorized as either arbitrary or functional according to early patterning processes in the *Drosophila* embryo. Spatio-temporal expression trajectories in virtual syncytial embryos were simulated via reaction-diffusion models. My *in silico* mutational experiments show that: 1) the average fitness decay tendency to successively accumulated mutations in ensembles of functional networks indicates the prevalence of positive epistasis, whereas in ensembles of arbitrary networks negative epistasis is the dominant tendency; and 2) the evaluation of epistatic coefficients of diverse interaction orders indicates that, both positive and negative epistasis are more prevalent in functional networks than in arbitrary ones. Overall, I conclude that the phenotypic and fitness effects of multiple perturbations are strongly conditioned by both the regulatory architecture (i.e. pattern of coupled feedback structures) and the dynamic nature of the spatio-temporal expression trajectories displayed by the simulated networks.

## Introduction

The relationship between the information contained in the genetic material and biological traits or functions (genotype-phenotype mappings (GPMs)) has represented a major challenge in biology, in which much research efforts have been devoted in the last decades [1]–[3]. Genotype-phenotype relationships had remained elusive until very recently, due to the lack of mechanistic understanding of the embryonary, physiological and metabolic action of the genes through their products. However, with the burgeoning of the “omics” disciplines (i.e. genomics and proteomics) and the advancement in genetic and molecular methodologies, a wealth deal of information has been gathered, providing us a very exciting picture on the structural and functional organization of genomes and proteomes [4]. For example, previous investigations on complex GPMs support the idea that phenotypic traits emerge as a result of the concerted action of many genes and their products, which tend to self-organize into regulatory networks (i.e. transcripional and cell signaling systems) below the scale of an entire genome or proteome [5]–[8]. These findings have thus revealed, to a large extent, the contents of these biological black boxes, suggesting that highly-modular regulatory networks are the mechanisms underlying complex GPMs.

One of the major difficulties toward the understanding and predictions of complex GPMs is the presence of a pervasive phenomenon referred to as epistasis, in which the phenotypic effects of genes are dependent on the genomic/genetic context in which they are embedded. In other words, non-linear correspondences between changes in the genotype and their phenotypic effects turn out to be inherent variational properties of highly dimensional GPMs. Due to these frequent context-dependent effects, epistasis has represented a major challenge for molecular genetics, population and quantitative genetics, as well as for evolutionary theory. These disciplines have strongly relied on black box-like concepts grounded mainly on genetic principles and their statistical descriptions as a means to give an explanation to GPMs. For instance, concepts such as genetic background and genetic architecture are at the heart of traditional frameworks aimed at addressing genotype-phenotype relationships in, for example, research programs focused on the etiology of diseases, animal and plant breeding, adaptation and speciation processes, etc (see [9] and references therein). In general terms, the classical paradigm of genetics has been grounded on static and abstract descriptions of gene effects and interactions, supported, basically, by an arsenal of statistical methodologies. Tradionally, investigations on genotype-phenotype relationships via statistical analysis have mainly resulted in linear/multilinear predictions, where no kind of mechanistic details or epigenetic phenomena have been possible to be accounted for in an explicit manner. However, with the fluorishing of systems biology, studies on GPMs have revealed that they are brought about dynamically via the action of complex molecular networks, wherein intricate functional dependencies among molecules and regulatory processes naturally emerge. This new research paradigm has provided substantial evidence supporting the idea that epistatic phenomena are pervasive at higher levels of biological organization above the genetic material. Thus, in addition to the characterization of genetic principles of GPMs, which have traditionally been encompassed under the umbrella of genetic architecture (i.e. ploidy, dominance, penetrance, expressivity, etc.), emphasis should be made on mechanistic descriptions and quantitative properties of molecular networks (i.e. regulation, control, dynamics, design principles, self-organization, emergence, etc).

A great deal of reviews on epistasis have been published, most of which addressing the issue from contrasting viewpoints. For example, a recent perspective provides an important view on epistatic phenomena and its diverse biological implications [10]. In that work, Phillips discusses functional, compositional and statistical epistasis. Functional epistasis is regarded as being relevant for molecular biology studies as a means to addressing possible interactions between genetic elements, which has been shown to provide insight into the structural and functional features of activation gene cascades [11]. On the other hand, Phillips suggests compositional epistasis as a new term intended to describe the classical usage of epistasis, in which special emphasis is made on genetic contexts and phenotypic effects of allelic substitutions. Finally, the statistical notion of epistasis attributed to Fisher [12] is defined as the deviation from additivity in linear statistical models, where the relationship between multilocus genotypes and phenotypic variation, at a population level, is not predictable under the assumption of independent gene activities. Statistical epistasis is a population property, and is a function of both allele frequencies and the biological interaction among genes [13], [14]. It can be argued that these concepts are only useful for descriptive purposes of epistatic phenomena, since they rely on statistical inferences that aimed at revealing genotype-phenotype relationships based on correlations and regressions of traits values. Thus, as stated above, these approaches are not specially suitable fo gaining a mechanistic understanding of how genetic variants or mutations are dynamically translated into complex phenotypic traits (i.e. gene expression patterns arising during embryogenesis). In contrast to classical paradigms, however, recent studies have discussed and highlighted the pervasiveness and consequences of epistasis at organizational levels above the genetic material. For example, Moore and colleagues have discussed the relationship between biological (also referred to as functional or physiological) and statistical epistasis from the scope of molecular networks [15], [16]. They emphasize that epistasis is a natural component in the biomolecular interactions that drive transcription, translation and signal transduction; they introduce the concept of biological epistasis, which is meant to describe how physical interactions among proteins or other molecules impact the phenotype.

Several quantitative measures for assessing mutational interaction patterns have been proposed (see [17] and references therein). Epistatic interactions are usually evaluated with respect to fitness costs, and more specifically with regards to phenotypes that are presumed to be relevant for survival. Hence, under fitness considerations, epistasis has been evaluated with respect to global transcriptional profiles in the slime mold [18], metabolic fluxes in yeast [19], growth rates and biomass production in bacteria [20]–[23], replication rates in viruses [24], and life history traits in insects [25]. Importantly, these studies have revealed the contrasting presence of two types of non-linear mutational interaction patterns: 1) synergistic pattern (also known as negative or aggravating), wherein the harm caused by multiple mutations at the fitness level tend to be more severe than when considering their mutational effects separately (independent effects); and 2) antagonistic pattern (also referred to as positive or buffering), whereby mutations tend to buffer or compensate each other's effects, which results in a considerable reduction of their combined effects on fitness as they accumulate succesively.

In summary, the broad research agenda that has been devoted to studying the phenotypic and fitness effects of mutational perturbations has convincingly demostrated that epistasis is a counter-intuitive and pervasive phenomenon in multidimensional GPMs. In general, it has been deduced that this should be mainly because genes and their products interact dynamically in hierarchical non-linear regulatory systems. I thus agree with some authors in that complex GPMs and epistatic phenomena represent a big challenge for modern biology, and are required to be addressed in the context of molecular networks by means of systems approaches [15], [26], [27]. In this spirit, here I report results from a series of computational experiments on mutational interactions in developmental regulatory network models that are specially relevant for early *Drosophila* embryogenesis (i.e. segment patterning). Spatio-temporal expression trajectories (developmentally-relevant phenotypes) in ensembles of highly-modular (fully interconnected) networks exhibiting distinctive regulatory topologies, which are operative in the context of one-dimensional syncytial embryos, were simulated. To this aim, computational models of reaction-diffusion mechanisms based on ordinary differential equations (ODEs) were implemented over developmentally relevant time scales. I analyzed ensembles of networks exhibiting (functional) and lacking (arbitrary) patterning capabilitites resembling those of the GAP network in the *Drosophila* embryo (see Materials and Methods section). Random perturbations in the regulatory interaction parameters governing the expression dynamics of the networks were systematically induced. Epistatic interactions among multiple simulated mutations according to their hypothetical impacts on fitness were evaluated; being the fitness a function of phenotypic discrepancies between mutant and optimal, or reference, spatio-temporal expression trajectories. More specifically, it was evaluated in each ensemble of networks modeled the average tendency in the fitness decay as mutations were accumulated successively in the networks, and epistatic coefficients of second, third, fourth and fifth order.

The results of the *in silico* mutational experiments show that: 1) the average fitness decay tendency to successively accumulated mutations in ensembles of functional networks indicates the prevalence of positive epistasis, whereas in ensembles of arbitrary networks negative epistasis is the dominat tendency; and 2) the evaluation of epistatic coefficients of diverse interaction orders indicate that, both positive and negative epistasis are more prevalent in functional networks than in arbitrary ones. Overall, I conclude that the phenotypic and fitness effects of multiple perturbations are strongly conditioned by both the regulatory architecture (i.e. multiple coupling of basic feedback motifs) and the dynamic nature of the spatio-temporal expression trajectories displayed by the simulated networks. These simulation results are discussed in the light of important themes of investigation, such as the relationship between the complexity of regulatory networks and the combinatorial effects of multiple mutational perturbations, as well as evolutionarily correlated responses.

## Materials and Methods

### Epistasis in the Context of Developmental Regulatory Networks

Unlike the classical conception of epistasis, which has been widely described and discussed in genetic terms (see for example [10], [17], [28]), the ideas developed below strongly rely on a mechanistic understanding of developmental networks and their quantitative properties. Specifically, my arguments will be explicitly grounded on both the structural and functional organization of transcriptional regulatory networks. Importantly, I will make special emphasis on: 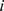) the regulatory schemes (i.e. operational rules such as feedforward/feedback structures) and 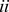) associated control parameters (mutable network properties defined in biochemical or biophysical terms) that determine the range of possible spatio-temporal expression trajectories accessible to the networks. Following this line of arguments, it is implicitly stated that the correct functioning of regulatory networks resulting in the reproducibility and stability of developmental processes can be assumed to be embedded in regulatory schemes and encoded in control parameters, which represent substantial sources of epistasis. Relying on these ideas, I thus introduce a definition of epistasis from a developmental systems perspective as a guideline for the interpretation of my simulation results: *Epistasis is the phenomenon in which the effects of diverse allelic configurations and mutational combinations propagate in a non-linear fashion through the regulatory schemes and control parameters governing the spatio-temporal expression trajectories of developmental networks. This should result in non-trivial correspondences between changes in the genotype and their phenotypic manifestation during, for example, embryonic multicellular patterning, hence bringing forth possible fitness costs (i.e. embryo viability)*.

### Regulatory Network Models

**Figure 1:**
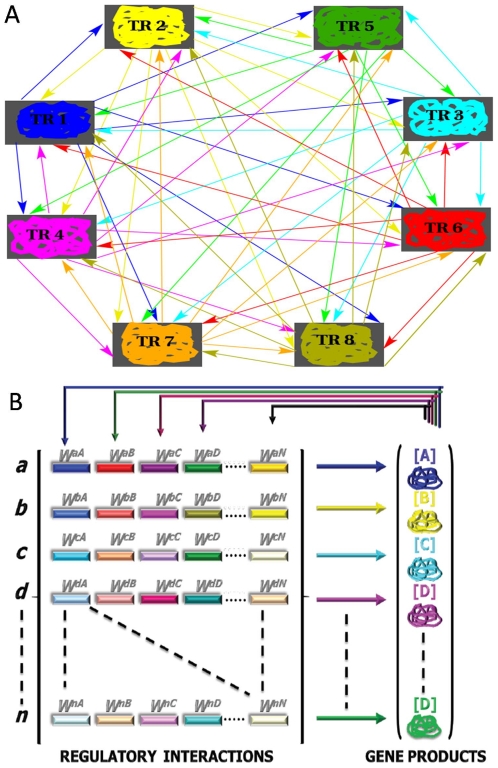
Regulatory Scheme and Parametric Structure of the Developmental Regulatory Network Models. Network models viewed as in A (regulatory scheme or topology) and B (parametric structure) can be thought of as a macroscopic approximation to the complex molecular interactions (i.e. DNA-protein binding) taking place during a transcriptional regulatory process; in this study such an approximation was made in order to modeling patterning networks in the context of one-dimensional syncytiums. This coarse-grained representation accounts for aggregated regulatory parameters summarizing the overall transcriptional effect of many individual binding sites arranged in complex cis-regulatory sequences. Thus, cross-regulatory interactions among transcriptional regulators are assumed to be captured in the 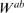 elements of the regulatory matrix (B), which can be thought of as a regulatory genotype defined in biochemical terms (biochemotype). Panel A illustrates a fully-interconnected regulatory topology encompassing 8 TRs, and 56 cross regulatory interactions (autoregulatory patterns are not shown). TR X indicates a transcriptional regulator X, and arrows represent functional dependencies among regulators, which are parameterized via the regulatory matrix (B). A 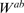 element in the matrix can assume any value ranging in 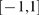, indicating negative or positive regulatory effects; this is the manner in which +/− feedback motifs are encoded in the matrix. This matrix of regulatory parameters is propagated dynamically via biochemical reactions within each nucleus modeled, 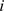, determining in this way the component of protein synthesis dynamics in the network models.

Mutational interactions in ensembles of fully interconnected networks encompassing configurations with 5, 6, 7 and 8 transcriptional regulators (TRs) were explored, which were categorized as either functional or arbitrary networks (see Figure 1A and Figures S4A-S4D). To this aim, reaction-diffusion models based on ODEs were implemented, which have been employed succesfully to reproduce early patterning processes and infer possible regulatory changes underlying mutant expression trajectories in the *Drosophila* embryo [29]–[32]. These models provide reasonable macroscopic representations of transcriptional regulatory networks in virtual embryos. It is worth mentioning that abstract unicellular versions of these models relying on Boolean rules have been widely implemented in previous studies, as a first attempt to capture general principles and emergent properties of regulatory networks. For instance, such coarse-grained network models have been used to shed light on evolutionary capacitors [33], robustness and neutral networks of genotypes [34], the role of feedback loops for the coexistence of robustness and fragility [35], and for assessing the implications of sexual reproduction in the evolutionary dynamics of robustness and negative epistasis [36]. My modeling approach, although being more biologically realistic, also makes macroscopic abstractions on molecular processes. However, unlike the models mentioned above, my modeling approach allowed to simulate and track the continuous trajectories of expression patterns in one-dimensional virtual syncytial embryos, over developmentally relevant time scales. Moreover, my computer experiments were based on quantitative expression data that have been employed for guiding pioneering computational studies on developmental pattern formation (see Figure S1). More specifically, this study was inspired in the GAP regulatory network that is deployed during early stages of *Drosophila* embryogenesis [29], [37]. In particular, functional network configurations were required to be capable of reproducing spatio-temporal dynamics similar to those reported for the GAP network (see Figures S2A–S2D). For example, the GAP network realizes diverse patterning tasks manifested in alternating and overlapping expression domains, sharp domain boundaries, and spatial shifts of expression domains along the anterior-posterior axis of the developing embryo [29], [37]. On the other hand, arbitrary network configurations were not required to exhibit biologically relevant patterning properties. They, instead, display either trivial or highly disordered expression trajectories characterized by uniformly distributed and spike-like expression domains (see Figures S3A–S3D). For each network configuration class (ranging between 5–8 TRs) it was constructed an ensemble of 15 different networks, categorized as either functional or arbitrary according to their patterning capabilitites mentioned above (see Supporting Information, Methods S1–S4). The mathematical representation of these networks is grounded on coarse-grained approximations to biochemical reactions and diffusion dynamics. These models do not explicitly account for neither genes, nor mRNA species, but for protein products (TRs), for which a state vector is defined as:
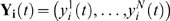
(1)which gives the concentration of TRs at time *t* in any nucleus *i*, whereas 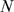 indicates the number of TRs in a regulatory network. The full dynamical system is represented in matrix form, with rows and columns accounting for nuclei and transcriptional regulators, respectively, as follows:
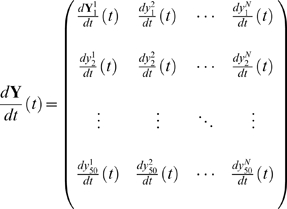
(2)


Where 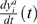 gives the time variation in the concentration of a transcriptional regulator *a* in nucleus *i*. Accordingly, every row of the matrix represents the dynamic expression of the network within nucleus *i*. One-dimensional syncytiums represented by a strip of 50 nuclei were modeled; in this way, these virtual developmental systems encompassed 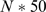 ODEs (with 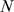 ranging between 5–8 TRs, thus amounting to 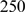
–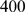 ODEs with associated configurations of control parameters ranging between 40–88) of the form:
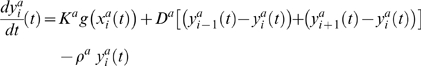
(3)


The three terms on the right-hand side indicate velocities of production, diffusion and degradation of a transcriptional regulator *a*, where the associated parameters 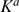, 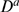 and 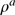 correspond to maximal production rate, diffusion and degradation rates, respectively. Additionally, it was assumed a transfer function 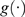 with sigmoidal-like saturation kinetics that depends on the regulatory interactions among TRs and their respective concentrations, which accounts for the expression dynamics (i.e. protein synthesis) of the regulatory network, defined as follows:
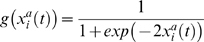
(4)


Detailed molecular processes such as transcription and translation are aggregated and assumed to be accounted for in this mathematical expression. The dynamic variable 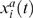 gives the total regulatory input exerted on a transcriptional regulator 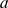, in a nucleus 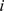 at time 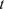, and is of the form:
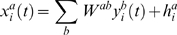
(5)


Here, the parameter 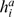 stands for regulatory inputs, of maternal origin, to every transcriptional regulator 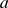 in each nucleus 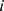. The interaction matrix 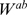 defines a network topology, and encapsulates in quantitative terms the strength of regulatory interactions among TRs, as well as the nature of such interactions (repressing/activating interactions; see Figures S4A-S4D for instances of functional topologies). This interaction matrix encodes regulatory information that is deployed dynamically by the networks through biochemical reactions; hence, this repository of analog information can be thought of as representing a regulatory genotype defined, somehow, in biochemical terms (see Figure 1B). The information contained in this matrix determines a network's capacity of finely controlling the spatio-temporal organization of expression domains along the strip of nuclei modeled (patterning capabilities). Thereby, I concentrated on this regulatory interaction matrix as the target for systematic perturbation analysis in the network models. It is worth noting that a mutation in any regulatory site 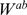 can be the result of a change in a *cis*-regulatory region of the gene 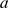 to which the regulator 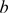 binds, or a change in the coding sequence for the regulator 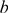 affecting a protein domain that binds to a *cis*-regulatory region of the gene 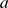. Such simulated mutations may result in two possible regulatory effects: 1) quantitative changes in regulatory interactions among TRs, or 2) rewiring of the operational rules of the network manifested in transitions between feedback regimes among TRs, such as (−) 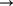 (+) or (+) 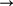 (−) feedback. Here I restricted the simulated mutational effects to the first case. It should be noted that these network models, and more specifically the regulatory interaction matrices they contain, do not explicitly consider the presence of individual regulatory sequences, but they can be thought of as macroscopic approximations to more precise models (i.e. based on thermodynamic principles) accounting for the activity of detailed regulatory sites distributed along an enhancer sequence (see Figure 1B). Therefore, the function and structure of enhancer sequences are somehow incorporated into the regulatory interaction matrix, 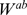, accounting for their averaged activities, and thus allowing for the establishment of net regulatory dependencies among TRs. Furthermore, it was assumed that the regulatory mechanisms modeled represented suitable (macroscopic) approximations to the underlying molecular mechanism, thus, altenative network models were not explored. For example, within the context of this coarse-grained model of transcriptional regulation, the sigmoidal function 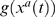 can only be regarded as a hypothesis on the modulation of the regulatory effect of one regulator according to the presence or absence of others. Such effects may range from synergistic potentiation to complete abolition of regulatory effects. In this function, cooperative effects may be representable by a large value of an appropiate 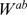, leading to a steep sigmoidal similar to a higher order Hill function. For further details on computational approaches see Supplementary Material (Methods M1 and M4).

## Results

### Evaluation of Averaged Fitness Decay Tendencies: Mutational Trajectories

**Figure 2:**
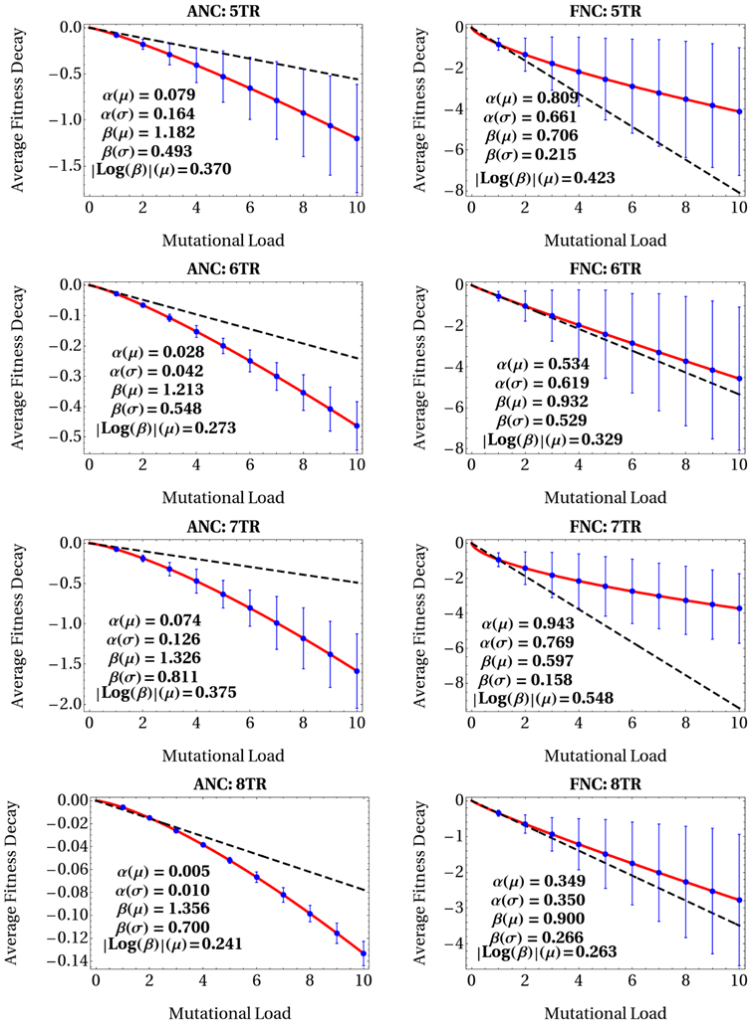
Average Mutational Trajectories with Respect to Fitness. Each plot summarizes the way in which successively accumulated mutations induce a fitness decline over each ensemble of networks modeled (average mutational trajectory). Black (dashed) line indicates a fitness decline in the absence of epistasis, which summarizes idealistic multiplicative effects among mutations (non-epistatic mutational trajectory, 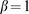). Red line indicates the calculated fitness decline. Average directionality of epistasis (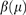) and standard deviation (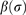), average strength of epistasis (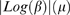), and average mutational sensitivity (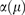) and standard deviation (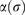) were evaluated. A legend above a graphic “ANC: X TRs” reads Arbitrary Network Configuration with X Transcriptional Regulators (X = 5–8). Similarly, a legend “FNC: X TRs” reads Functional Network Configuration with X Transcriptional Regulators.

The first *in silico* mutational experiment was performed with the aim of characterizing the statistical behavior of the developmental propagation of combined mutational effects, and their possible impacts at the fitness level. Speciffically, I explored the way in which succesively accumulating mutations in a virtual embryo carrying a reference regulatory network impinged on the expression trajectories. A maximum of 10 mutations in each of the 15 networks of an ensemble were induced, whose fitness costs were evaluated as they accumulated; 2000 random mutational combinations were generated in each ensemble analyzed (for details on fitness calculations see Methods S2 and S3). The resulting average fitness decay tendency for each reference network, within an ensemble, was fit to the equation 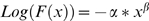, where 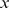 stands for the number of accumulated mutations, and the parameters 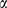 and 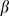 indicate average mutational insensitivity of the system, and average directionality of mutational interactions with respect to fitness, respectively [38]. Mutational effects on fitness reflecting independency would be observed for 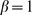; for a 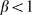 each successive mutation would tend to delay the fitness decline (positive epistasis); and for a 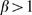 each additional mutation would tend to accelarate the fitness decline (negative epistasis). The analysis of mutational trajectories (see Figure 2) over each ensemble of networks modeled show that the average tendency in the form successively accumulated mutations induce a fitness decline in arbitrary networks is clearly indicative of negative epistasis (red lines). In contrast, in functional networks mutations tend, on average, to compensate each other's effect as they accumulate, indicating positive or buffering epistasis. As mentioned above, the coefficients 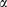 and 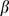 capture the statistical behavior of the ensemble of networks modeled with respect to mutational insensitivity and directionality of mutations. Thus, they are important estimators that allow the comparisson of average effects between ensembles of arbitrary and functional networks, as well as between ensembles of networks exhibiting differing topological (structural) complexity. For example, the analysis indicates that the average mutational insensitivity in ensembles of arbitrary networks is greater than in those of functional ones (indicated by 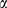 values closer to zero), thus corroborating the existence of a tight correlation between directionality of epistasis and average insensitivity [39]. It is also important to note that, comparatively, the average strength of epistasis (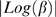) turned out to be larger in esembles of functional networks than in those of arbitrary networks. Finally, despite having found general tendencies regarding the statistical mutational behavior of the regulatory networks, no systematic relationship was observed between increasing network complexity and the epistatic nature of the regulatory networks. This observation contrasts with those results obtained in a recent study on epistatic interactions in simple network models [40], suggesting the existence of a general correlation between the average directionality of epistasis and network complexity (see below).

Note in Figure 2 that fits of the equation 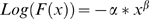 (red line) to average data (blue points) were remarkable accurate, and that departures from theoretical predictions of non-epistatic trajectories ( 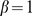, black dashed line) were significative for each ensemble analyzed. It is also interesting to note that the variability around average Log-scale fitness (indicated by blue bars) tended to increase as mutations were accumulated in the networks, suggesting a high probability for drastic epistatic fluctuations to be observed in the data. More importantly, the variability around average data were consistently larger in ensembles of functional networks than in those of arbitrary networks. This observation is congruent with an intriguing finding concerning the frequency with which individual mutational trajectories undergo transitions between positive and negative epistatic regimes. For example, at a finer level of the analysis it was observed that as indivual mutational trajectories in ensembles of functional networks were evaluated, abrupt changes in the directionality of epistasis (from positive to negative or negative to positve directions) were surprisingly frequent. However, in the case of ensembles of arbitrary networks such shifts between epistatic regimes were practically unattainable, and instead, individual trajectories were predominatly stable in their directionalities. Changes in the directionality of mutational interactions with respect to fitness are indicative of a particular epistatic phenomenon referred to as sign epistasis [41], which is thought to be a key factor shaping complex evolutionary trajectories in the fitness landscape. In the case of my simulation results, the fact that sign epistasis is a natural property realizable in functional networks alone would be consistent with the idea that their evolutionary dynamics in the fitness landscape may take place over very rugged topographies, with multiple adaptive peaks separated by small valleys.

### Evaluation of Epistatic Spectra: Distribution of Epistatic Coeficients

The first analysis provides interesting insight into average mutational trajectories with respect to fitness (fitness decline tendencies). However, such methodology is not appropriate to uncovering the distribution of combined mutational effects with respect to fitness, given that positive and negative epistasis tend to cancel each other out, on average. More specifically, this analysis is not meant to uncovering the full spectrum of interaction patterns among mutations. That is way the estimation of epistatic spectra prove to be a more suitable approach for the exploration of both the coexistence of positive and negative epistasis, and their possible fluctuations around a mean value. Therefore, in an attempt to reveal the absence or presence of such statistical regularities, a conventional non-scaled measure of epistatic interactions (see [17]) was implemented, which is founded on a null model assuming independence of mutational effects (multiplicative model):
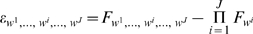
(6)


**Figure 3:**
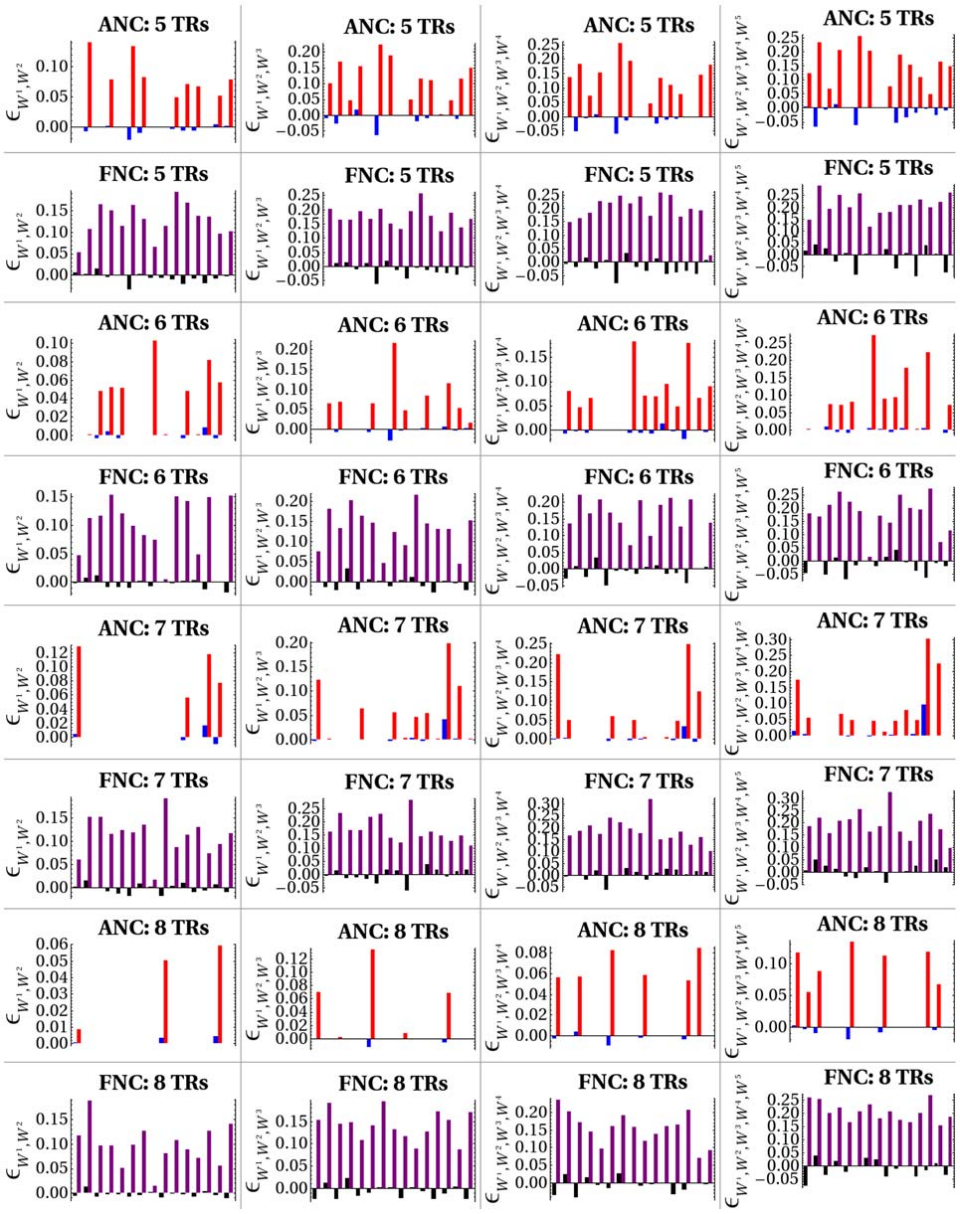
Matrix of Barplots on Mutational Spectra. Mutational experiments were categorized according to either arbitrary or functional network configurations. Each row in this matrix summarizes results from mutational experiments performed in an ensemble of either arbitry or functional network configurations, whereas each column gives the order of the epistatic coefficient analyzed, increasing from second (left most column) to fifth order (right most column). Height of blue and black bars corresponds to average values of epistatic interactions (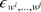 values) over each ensemble of networks simulated. Height of red and purple bars indicates standard deviations. Mutational experiments encompassing 2, 3, 4 and 5 mutational hits are organized in column form. A legend above a graphic “ANC: X TRs” reads Arbitrary Network Configuration with X Transcriptional Regulators (X = 5–8). Similarly, a legend “FNC: X TRs” reads Functional Network Configuration with X Transcriptional Regulators.

Here, 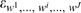 defines a mutational interaction coefficient that depends on: the fitness of a virtual embryo carrying a multiple mutant regulatory network with 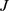 perturbations, 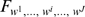, and the product of individual fitness values associated to an embryo carrying a single mutant network, 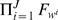. The subindex 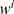 stands for a mutational hit in any element 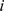 of the regulatory interaction matrix, 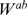 (for details on fitness calculations see Methods S2 and S3). This model assumes that the expected distribution of combined mutational effects on fitness is centered around zero (neutral mutational effects), which, in our case, would be a clear indication of the absence of functional dependencies among the nodes within a network. Hence, any deviation from neutrality would be indicative of non-linear mutational interactions with respect to fitness. In this computational experiment, the spectrum of mutational interactions for each reference network modeled within a given ensemble analyzed was characterized under combinations of multiple perturbations. Epistatic coefficients of second (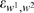), third (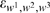), fourth (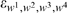), and fifth (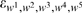) order were evaluated. Each network of an ensemble was subjected to 1000 systematic rounds of mutational perturbations in order to evaluate the distribution of each epistatic coefficient. The results are illustrated in Figure 3, which is a matrix of barplots summarizing mean and standard deviations of epistatic coefficients (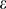). The analyses reveal intriguing general tendencies in the manner in which mutational perturbations interact: 1) Departures from neutrality (as indicated by the height of blue and black bars) tend to be more frequent in ensembles of functional networks than in those of arbitrary ones, and for any level of network complexity analyzed, thus indicating the ubiquity of non-linear mutational interactions in functional networks. 2) The intensity of epistatic interactions, either positive or negative, tend to be stronger in functional networks than in arbitrary ones. 3) The variability of epistatic coefficients shows a surprising homogeneous tendency in ensembles of functional networks, whereas in ensembles of arbitrary networks the variability shows a remarkably fluctuating tendency. 4) In ensembles of arbitrary networks the frequency of observed non-linear mutational interactions tend to decline dramatically as the structural complexity of the networks increases, whereas in functional networks the richness of epistatic interactions is maintained and tend to be surprisingly homogeneous. It is also important to note that, despite of the presence of substantial non-linear mutational interactions in ensembles of functional networks, no clear tendency can be drawn with respect to the prevalence of one particular type of epistasis. The results show, instead, that mutations tend to compensate or reinforce their effects in surprinsingly equal proportions, thus indicating that the epistatic architecture (i.e. statistical behavior of combined mutational effects with respect to fitness) of functional networks turn out to be rather complex. Further, it is worth emphasizing that these experiments reveal general tendencies in the manner in which multiple mutations interact that can be extremely weak. Thus, it follows that the biological implications of these predicted interactions might be, in principle, impossible to be traced experimentally. For example, note from Table 1 that the maximun and minimun average values observed within each ensemble analyzed tend to be extremely weak. Nevertheless, one can appreciate that, in general, the intensity of epistatic interactions is stronger in ensembles of functional networks than in those of arbitrary ones; note also that the range of variation is larger in ensembles of functional networks. To gain a clearer idea on the statistical significance of the strength of epistatic interactions in ensembles of functional and arbitrary networks, sign tests were run. Table 2 provides information about the number of networks in each mutational experiment performed in a given ensemble that exhibited epistasis (either positive or negative) significantly different from 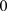. The results of the tests confirm that epistasis is far more intensive in ensembles of functional networks, and more importantly, that it is more frequent to find epistasis in functional networks than in arbitrary ones as the mutational load increases.

**Table 1:** Average Epistatic Coefficient.

	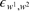	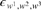	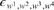	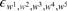
ANC: 5TR	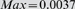 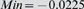	0.0172 −0.0628	0.0081 −0.0590	0.0111 −0.0670
FNC: 5TR	0.0150	0.0189	0.0326	0.0418
	−0.0350	−0.0625	−0.0786	−0.0915
ANC: 6TR	0.0081	0.0071	0.0135	0.0081
	−0.0040	−0.0289	−0.0198	−0.0098
FNC: 6TR	0.0112	0.0327	0.0324	0.0423
	−0.0191	−0.0270	−0.0501	−0.0682
ANC: 7TR	0.0172	0.0415	0.0335	0.0957
	−0.0099	−0.0042	−0.0080	−0.0034
FNC: 7TR	0.0148	0.0399	0.0292	0.0504
	−0.0198	−0.0594	−0.0608	−0.0435
ANC: 8TR	0.0042	0.0000	0.0038	0.0224
	0.0000	−0.0138	−0.0101	−0.200
FNC: 8TR	0.0124	0.0227	0.0261	0.0390
	−0.0115	−0.0245	−0.0429	−0.0756

Maximun and minimun values for each average epistatic coefficient ( 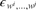) evaluated over an ensemble encompassing 15 networks, categorized as either arbitrary or functional. ANC: XTR indicates arbitrary network configurations with X Transcriptional Regulators. FNC: XTR indicates functional network configurations with X Transcriptional Regulators.

**Table 2:** Percentage of Epistatic Networks.

	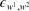	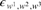	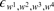	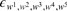
ANC: 5TR	1/15	2/15	2/15	4/15
FNC: 5TR	4/15	5/15	9/15	11/15
ANC: 6TR	0/15	0/15	0/15	0/15
FNC: 6TR	4/15	4/15	8/15	8/15
ANC: 7TR	0/15	0/15	1/15	1/15
FNC: 7TR	4/15	5/15	8/15	9/15
ANC: 8TR	0/15	0/15	0/15	0/15
FNC: 8TR	1/15	5/15	5/15	8/15

Percentage of Networks with Average Epistatic Coefficients Significantly Different from 0. Statistical significance was evaluated by means of sign tests (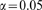). Each matrix entry gives the percentage of networks exhibiting epistasis (either positive or negative) significantly different from 0, under different mutational conditions: epistatic coefficients of two (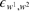), three (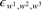), four (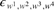) and five (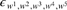) orders. ANC: XTR indicates arbitrary network configurations with X Transcriptional Regulators. FNC: XTR indicates functional network configurations with X Transcriptional Regulators.

### Regulatory Schemes and Spatio-Temporal Propagation of Combined Mutational Effects

**Figure 4:**
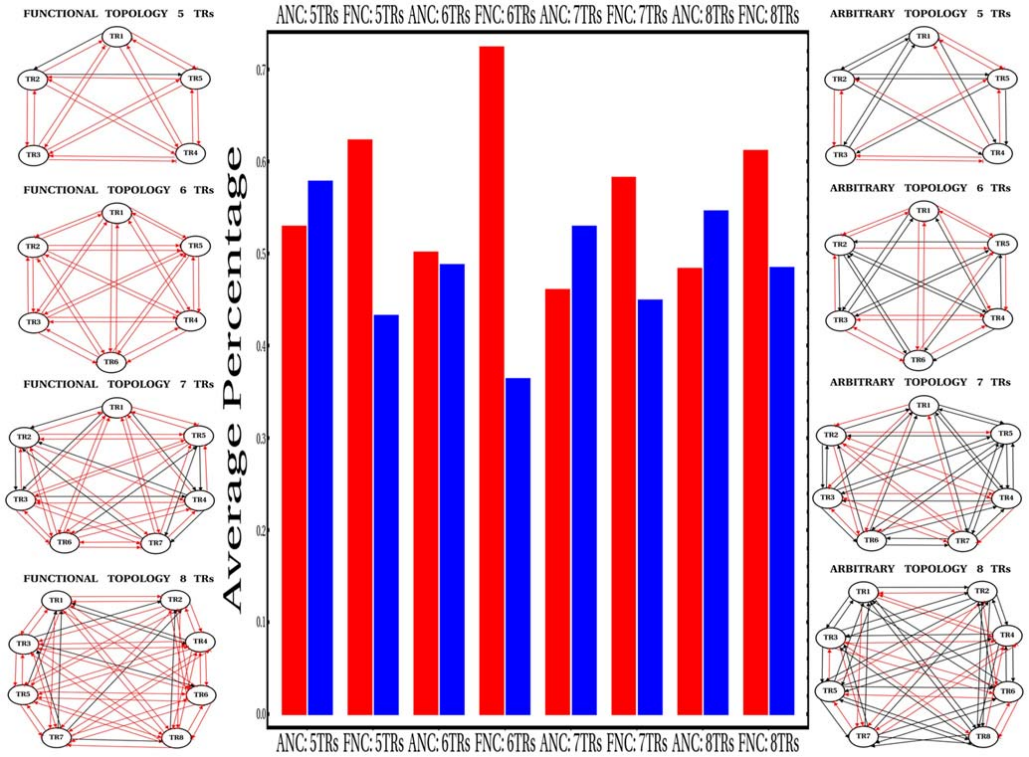
Statistics on the Regulatory Architecture and Representative Topologies of the Ensembles of Networks. Central panel provides bar plots illustrating average percentages of negative regulatory interactions (red bars) and basic negative feedback motifs (blue bars), over each ensemble of networks analyzed. Mann-Whitney tests were run in order to evaluate statistical significance between means of ensembles encompassing functional and arbitrary networks, for a given network configuration ranging between 5–8 TRs. P-values 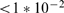 were found in the set of tests evaluating differences in average percentages of negative regulatory interactions, whereas P-values 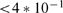 were obtained in the set of tests evaluating differences in average percentages of basic negative feedback motifs. In the horizontal axis of central panel a mark “ANC: X TRs” reads Arbitrary Network Configuration with X Transcriptional Regulators (X = 5–8). Similarly, a mark “FNC: X TRs” reads Functional Network Configuration with X Transcriptional Regulators. Representative topologies are shown for ensembles of functional (left panel) and arbitrary (right panel) networks, which were assembled according to the frequency of negative (red arrows) and positive (black arrows) regulatory interactions observed in each ensemble. Each topology shown does not illustrate autoregulatory patterns; only cross-regulatory interactions between TRs are shown.

Here, it is shown that heavy emphasis should be made on the structural and functional organization of regulatory networks in order to gain a mechanistic understanding of how the information contained in the genotypes is dynamically decoded into phenotypes, and to infer the possible impacts of this mapping at the fitness level. In particular, I previously referred to regulatory schemes as operational rules, such as feedback/feedforward control structures, governing the dynamic behavior of the networks. The local structural organization of molecular networks, which is composed of motifs, has been the focused of extensive theoretical investigation [7], [42]. For example, negative feedback regulation has been proposed as a mechanism capable of efficiently modulating the phenotypic effects of perturbations. This regulatory motif has been frequently regarded as a mutational buffering mechanism underlying robust properties of transcriptional and signaling networks [43], [44]. Alternatively, positive feedback has often been associated with amplification of perturbations [45], which may eventually account for some evolvable properties of the networks. Moreover, results from a pioneering work combining simple dynamical models of regulatory networks with statistical genetic methods support the idea that different feedback structures may yield differential epistatic patterns [26]. These observations are intriguing in the light of my simulation results, because it is not clear at all whether the design principles of arbitrary networks differ considerably from those of functional networks, so as to give a reasonable explanation to those remarkable discrepancies in epistatic architectures observed. Therefore, simple design principles in each ensemble of networks were evaluated statistically. Particularly, the highly modular nature of the networks simulated permits the analysis of some regulatory features encapsulated in the interaction matrix, 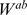, such as the density of negative (repressing) and positive (activating) interactions, as well as a detailed evaluation of basic feedback motifs that tend to be interlocked. In the first case, I consistently found that the average percentage of negative interactions were significantly larger in the ensembles encompassing functional networks than in those of arbitrary ones (Central panel in Figure 4, illustrated by red bars). Left and right panels in Figure 4 illustrate the topologies that turned out to be the most representative (according to the frequency of the +/− interactions) for the ensembles of networks simulated. From these two sets of topologies one can appreaciate that in ensembles of functional networks negative regulatory interactions (red arrows) prevail over positive interactions (black arrows). This simple observation suggests that the non-linear spatio-temporal propagation of combined mutational effects would require the presence of substantial repressing effects in the topology of functional developmental regulatory networks. On the other hand, given that these networks exhibit fully interconnected regulatory topologies (i.e. complete functional interdependencies among TRs) that are amenable to be decomposed into essential feedback motifs between pairs of TRs (see [29], [46]), one can thus analyze the basic regulatory architecture representative of each ensemble of networks modeled. To this aim, the average percentage of basic negative feedback motifs were calculated for each ensemble in this way: all possible different pairs of matrix elements 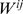 and 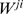 (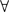 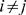) were assessed, which is given by 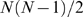, with N being the number of nodes in a given network configuration ranging between 5–8 TRs. Note that under this consideration, autoregulation patterns do not classify as feedback motifs. Then, the regulatory nature of a feedback motif (being positive or negative) was easily determined according to the parity of the number of negative interactions involved (i.e. −/− or +/+ define a positive feedback, and +/− or −/+ define a negative feedback). The results of the analysis are shown in Figure 4 (central panel, illustrated by blue bars), and indicate that a predictable under-representation of simple negative feedback motifs is observed in ensembles of functional networks (as opposed to arbitrary networks) given that such topologies exhibit a considerable excess of negative (repressing) interactions. Taken together, these results strongly suggest that both the regulatory nature of the interactions and the enrichment of simple building blocks, such as feedback motifs, in complex regulatory networks may affect the frequency with which contrasting mutational interaction patterns emerge. In this way, possible mechanistic insights on epistatic phenomena in highly-dimensional GPMs may be inferred by means of a detailed analysis of the design principles of the underlying networks [26], [40].

### Network Complexity vs Directionality of Mutational Interactions

**Figure 5:**
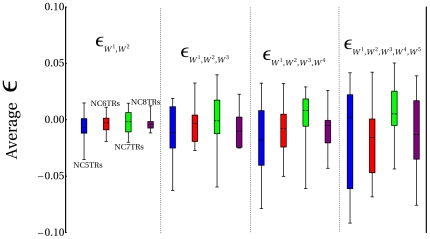
Epistatic Tendencies in Ensembles of Functional Networks. Box plots summarizing the statistical behavior (median and quantiles) of epistatic coefficients within ensembles encompassing 5 (blue), 6 (red), 7 (green) and 8 (purple) TRs. Epistatic coefficients ranging between 2 to 5 mutational combinations ( 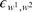 to 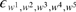) were evaluated for each ensemble of networks. Medians are indicated by horizontal dashed lines within each box. Variability around medians are indicated by whiskers. Vertical dashed lines separate groups of comparison. NC: XTR indicates network configurations with X Transcriptional regulators.

Previous analyses on average fitness decay tendencies were found not provide support to the presence of a deterministic relationship between increasing network complexity and the epistatic nature of the regulatory networks, as oppossed to those findings reported in [40]. To more clearly appreciate this observation, a simple statistical analysis was carried out in the data shown in Figure 3. In this case, I concentrated only on functional networks. For the ensembles of networks encompassing 5 to 8 TRs, box plots with mean values (calculated from 15 values in total for each ensemble analyzed) of epistatic coefficients of second, third, fourth and fifth order, were constructed. Figure 5 illustrates this set of box plots, with boxes colored in blue, red, green and purple corresponding to networks with 5, 6, 7 and 8 TRs, respectively. In the first place, the analysis indicates that, in general, as the number of mutations evaluated increases (from 2 to 5), the variability (indicated by the size of the boxes and the extent of the whiskers) in mean epistatic coefficients around the median, within each ensemble analyzed, tend to increase. However, no clear tendency in median values for each ensemble of networks were observed, which is in agreement with the analysis performed above. Now, in the case of comparisons between ensembles of networks with distinctive topologies, it is not possible to draw up a clear relationship regarding the structural complexity of a network and the overal tendency of epistatic coefficients. For example, from Figure 5 it is clear that when comparing median values (horizontal dashed lines in boxes) among ensembles, and for a given number of mutational combinations evaluated, no systematic behavior is observed with respect to changes in the overall tendency of epistasis. And this conclusion applies for any number of mutational combinations evaluated. If we were to observe in the data an indication of a systematic relationship between network complexity and the directionality of epistasis, we should then appreciate a clear tendency in the medians of the ensembles, being such tendency either increasing or decreasing as network complexity augments. Nevertheless, it could be argued that a significant tendency between directionality of epistasis and network complexity might only be appreciated by testing networks beyond certain degree of complexity, perhaps, networks exhibiting considerable differences in the number of nodes (TRs). It should be noted, however, that for the kind of highly modular (i.e. fully interconnected) developmental regulatory networks analyzed here, complexity is not only defined in terms of the number of TRs, but mainly in terms of the density of regulatory interactions and the coupling pattern of feedback structures. In this way, network complexity increases dramatically, and in ways that are not intuitive, as the number of component nodes (TRs) increases (see for example Figures S4A–S4D). Therefore, this series of *in silico* mutational experiments suggest that, in highly modular developmental regulatory networks the relationship between their structural complexity and the manner in which mutational combinations interact at the phenotypic and fitness level, does not seem to be a deterministic outcome of these systems. Rather, my simulation results strongly suggest that due to the complex regulatory architecture and spatio-temporal dynamics displayed by functional developmental networks, epistatic tendencies turn out to be unexpected emergent properties of these particular GPMs.

## Discussion

In this study, efforts were made with the aim of providing a mechanistic conceptualization on epistatic phenomena in the context of developmental regulatory networks. A series of computer experiments were performed in order to explore epistatic interactions among a wide spectrum of simulated mutational perturbations in ensembles of regulatory network models relevant for early *Drosophila* embryogenesis. On these grounds, epistasis is manifested as non-linear correspondences between mutational perturbations in the regulatory properties of the networks and their spatio-temporal expression trajectories. Epistatic effects were evaluated, ultimately, according to an intuitive fitness criterion represented by a mathematical function, being this dependent on phenotypic discrepancies between mutant and reference spatio-temporal expression trajectories.

The computational approach implemented in this study allowed for the systematic exploration of the mutational interaction space of model developmental regulatory networks. Importantly, this approach permitted a bounded excursion into the adaptive landscape of these regulatory systems, revealing the existence of remarkable differences between networks capable of accomplishing specific developmental patterning tasks, and those networks behaving arbitrarily. The major findings in this study can be summarized in the following points: 1) Analysis of average fitness decay tendencies to successively accumulated mutations showed that positive epistasis prevailed in ensembles of functional networks, whereas in ensembles of arbitrary networks negative epistasis was the dominat tendency. 2) Analysis of epistatic coefficients of diverse interaction orders showed that both positive and negative epistasis were more prevalent in functional networks than in arbitrary ones.

Despite having shown the prevalence of non-linear mutational interactions in functional networks, one may question whether the intensity of epistasis observed in these model networks may actually be biologically relevant. In the case we were to judge the relevance of these simulation results within an evolutionary context, the implications of weak mutational effects for the dynamics of adaption processes would not be clear at all. On this regard, several experimental studies have been devoted to assess the phenotypic effects and adaptive costs of induced single and combined mutations in different species [47]–[49]. Nevertheless, these studies are limited by the sensitivity of the experimental methodologies, which lack the power of detecting small mutational effects and have, instead, proven to be more effective at discovering mutations of major phenotypic impacts. However, as disscused in recent works on evolutionary systems biology (see [27], [50] and references therein), computer simulations of model genotype-phenotype mappings have proven to be a powerful approach aimed at estimating a wide spectrum of mutational combinations, their phenotypic effects, and hypothetical fitness costs. Most importantly, these *in silico* approaches are specially suitable for evaluating small mutational effects that are impossible to be assessed via current experimental methodologies, and whose biological impacts might become evident only at the long evolutionary run.

Intriguingly, the presence of significant differences in the design principle of functional and arbitrary networks were revealed, providing in this way a mechanistic explanation to the remarkable differences in epistatic architectures observed. Specifically, a consistent over-representation of negative regulatory interactions in the topology of functional patterning networks was found, implying the presence of abundant cross-repressing interactions between pairs of TRs. This resulted, in turn, in the coupling of multiple positive feedback motifs. Interestingly, these features have been reported as being fundamental design principles of the GAP network underlying the particular spatio-temporal trajectories of the system [29], [46]. Taking together, all these numerical experiments have yielded results that are worth discussing in the light of important themes of investigation. In what follows, I thus place the discussion of my simulation results in the context of specific topics.

### Dynamic Nature of Expression Patterns Constrains the Combined Effects of Mutational Interactions

The particular expression dynamics displayed by functional networks have been shown to be accessible only under the presence of substantial negative interactions in their regulatory topologies, thus implying the under-representation of basic negative feedback motifs between pairs of transcriptional regulators. Moreover, the evaluation of epistatic interactions via a null model assuming independecy of mutational interactions showed that the distributions of epistatic coefficients for arbitrary networks tended to be centered around zero. Importantly, this is clear evidence of the abscence of specific regulatory dependencies among the nodes within arbitrary networks, which is an operative condition incongruent with biologically relevant emergent behaviors. By contrast, for functional networks the distributions tended to cover a wide accessible range of epistatic patterns (positive and negative epistasis in diverse intensities), supporting the existence of specific regulatory dependencies within this type of networks. These results provide substantial numerical evidence supporting the following idea: since arbitrary networks were not required to fulfill specific patterning tasks, the degree of particular regulatory dependencies (i.e the density of repressing interactions) among the nodes within the networks turned out to be irrelevant. Consequently, an overall tendency in the distributions of mutational coefficients centering around zero would be expected to arise. In contrast, the degree of regulatory dependencies among the nodes within the functional networks would be predictably strong, since a remarkable coordinated regulatory action, in time and space, among TRs is required, so as to accomplish specific patterning tasks as observed in *Drosophila* embryos (i.e. alternating and overlapping expression domains and sharp domain boundaries). Strong and accurate functional dependencies in realistic patterning networks, which have likely been fine-tuned over evolution, should impose severe constraints in the manner in which combined mutational effects propagate dynamically during development. Taking together, these observations lead me to hypothesize that pervasive, and contrasting, epistatic interactions in actual developmental regulatory networks should be expected to arise, and that they may be naturally encoded in both their coupling pattern of feedback structures (regulatory architecture) and the complex emergent spatio-temporal expression trajectories. Following this idea, it is interesting to note that recent studies that aimed to explain the underlying mechanistic basis of robust developmental networks suggest that, robustness is highly dependent on the dynamic nature (either transient or stationary) of expression patterns [51]–[53]. Therefore, it is very likely that the regulatory processes (i.e. dynamic coupling of +/− feedbacks) underlying most developmental patterning events constitute substantial sources of epistasis, and thence, the robustness and evolvability of the underlying regulatory networks would be a clear manifestation of this.

### Epistasis as an Evolutionarily Correlated Response

The dynamic coupling of multiple +/− feedback loops displayed by the GAP network during early stages of *Drosophila* embryogenesis has been shown to be the mechanistic basis underlying the particular spatio-temporal expression trajectories (i.e. alternating and overlapping sharp expression domains) of the segmentation genes [29], [37], [54]). Such dynamic regulatory features have probably been shaped by strong selective pressures, given that some phases of the segmentation process have been reported to be remarkably conserved among closely related species of insects [55], [56]. These observations raise interesting questions regarding possible implications of the regulatory architecture of the segmentation network for the evolution of correlated responses, or biological spandrels (evolutionary byproducts) as proposed by SJ Gould [57]. On this regard, previous theoretical investigations have provided interesting insights on regulatory and emergent properties of transcriptional networks deployed during early *Drosophila* embryogenesis, which are particularly suggestive of the existence of biological spandrels. For example, a recent study suggests that very sharp transcriptional responses during *Drosophila* segment patterning may only be attainable under very specific regulatory conditions, namely, via transcriptional regulation dominated by a fine combination of activating/repressing regimes [51]. These authors showed, via mathematical modeling and computer simulations, that such regulatory conditions may account for robust responses in the face of parameter changes in the underlying transcriptional network. This observation led them to hypothesize that robust expression patterns may evolve as a by-product of direct selection for transcriptional switches. Similarly, in a seminal work on thermodynamic modeling of the transcriptional control of the *hunchback* gene by the morphogen Bicoid, Gibson showed that inevitable trade-offs between threshold widths and locations of maximal transcriptional responses in the *Drosophila* embryo are expected to ocurr, leading, naturally, to the emergence of epistasis and pleiotropic effects [58]. Based on these observations and my simulation results, I hypothesize that direct selection pressures (i.e. stabilizing selection) on the regulatory architecture of the networks underlying the complex spatio-temporal organization of the segmentation process might have caused evolutionarily correlated responses in epistatic architectures. This evolutionary scenario, for example, may have led to the emergence of by-products encompassing both positive (buffering) and negative (aggravating) epistatic interactions with respect to fitness components (i.e. embryo viability).

### Final Remarks

The proposed interpretation of epistatic phenomena in the context of molecular developmental mechanisms may represent an important step toward bridging the conceptual divide between classical and modern biological disciplines. Specifically, attempts have been made to reconcile the field of quantitative genetics and the more engineering-influenced field of molecular systems biology. Thus, it is hoped this work motivates future multidisciplinary studies between diverse emergent fields, such as systems quantitative genetics [59] and evolutionary systems biology [27], [50]. On the other hand, as further continuation of this work, it would be important to evaluate whether in larger and biologically realistic functional networks, such as metabolic and signal transduction *metanetworks*, the directionality of epistasis scales deterministically with increasing network complexity; being network complexity defined in both structural and functional terms. Finally, it remains to be seen whether my numerical findings about sign epistasis can be supported by experimental evidence from mutational studies in the *Drosophila* embryo and other model organisms, such as nematode worms and zebra fish. Nevertheless, I suspect that epistatic phenomena should be pervasive in regulatory systems that have evolved a fine coupling of multiple feedback structures allowing for ultrasensitive responses, such as those switch-like dynamics that are defining features of most signaling pathways and transcriptional regulatory networks partitioning the embryo into discrete expression domains.

## Supporting Information

Figure S1 GAP Network: Wild Type Spatio-Temporal Expression Trajectories. Snapshots of wild type expression domains of the GAP network at different time points during early Drosophila embryogenesis. This network encompasses transcriptional regulators of maternal and zygotic origin, such as Bicoid, Caudal, Hunchback, Krupel, Giant, Knirps, Tailles, which get involved in complex patterns of cross-regulation, and subsequently provide regulatory inputs to downstream expression cascades of the segmentation network. C13 to T5 indicate time points along the expression trajectory of the network, during early Drosophila embryogenesis. This time window covers early to late stages of cleavage cycle (nuclear division) 14A of the Drosophila blastoderm. C13 = 40.250 mins; T1 = 53.925 mins; T2 = 60.175 mins; T3 = 66.425 mins; T4 = 72.675 mins; T5 = 78.925 mins. Expression Data from [29], [37]
(0.03 MB PDF)

Figures S2 Representative Spatio-Temporal Expression Trajactories: Functional Networks.
(0.30 MB PDF)

Figures S3 Representative Spatio-Temporal Expression Trajactories: Arbitrary Networks.
(0.29 MB PDF)

Figures S4 Exemplar Network Topologies: Functional Networks.
(0.19 MB PDF)

Method S1 (0.03 MB PDF)

Method S2 (0.04 MB PDF)

Method S3 (0.05 MB PDF)

Method S4 (0.02 MB PDF)

Tables S1 (0.06 MB PDF)

Tables S2 (0.06 MB PDF)
