# Supplementary material for: A Developmental Systems Perspective on Epistasis: Computational Exploration of Mutational Interactions in Model Developmental Regulatory Networks
Source: PLoS One. 2009 Sep 7;4(9):e6823. doi: 10.1371/journal.pone.0006823 (PMC2734181; doi:10.1371/journal.pone.0006823)

A Developmental Systems Perspective on Epistasis: Computational Exploration of Mutational Interactions in Model Developmental Regulatory Networks

Jayson Gutiérrez

Supporting Information Figure S1.

GAP Network: Wild Type Spatio-Temporal Expression Trajectories

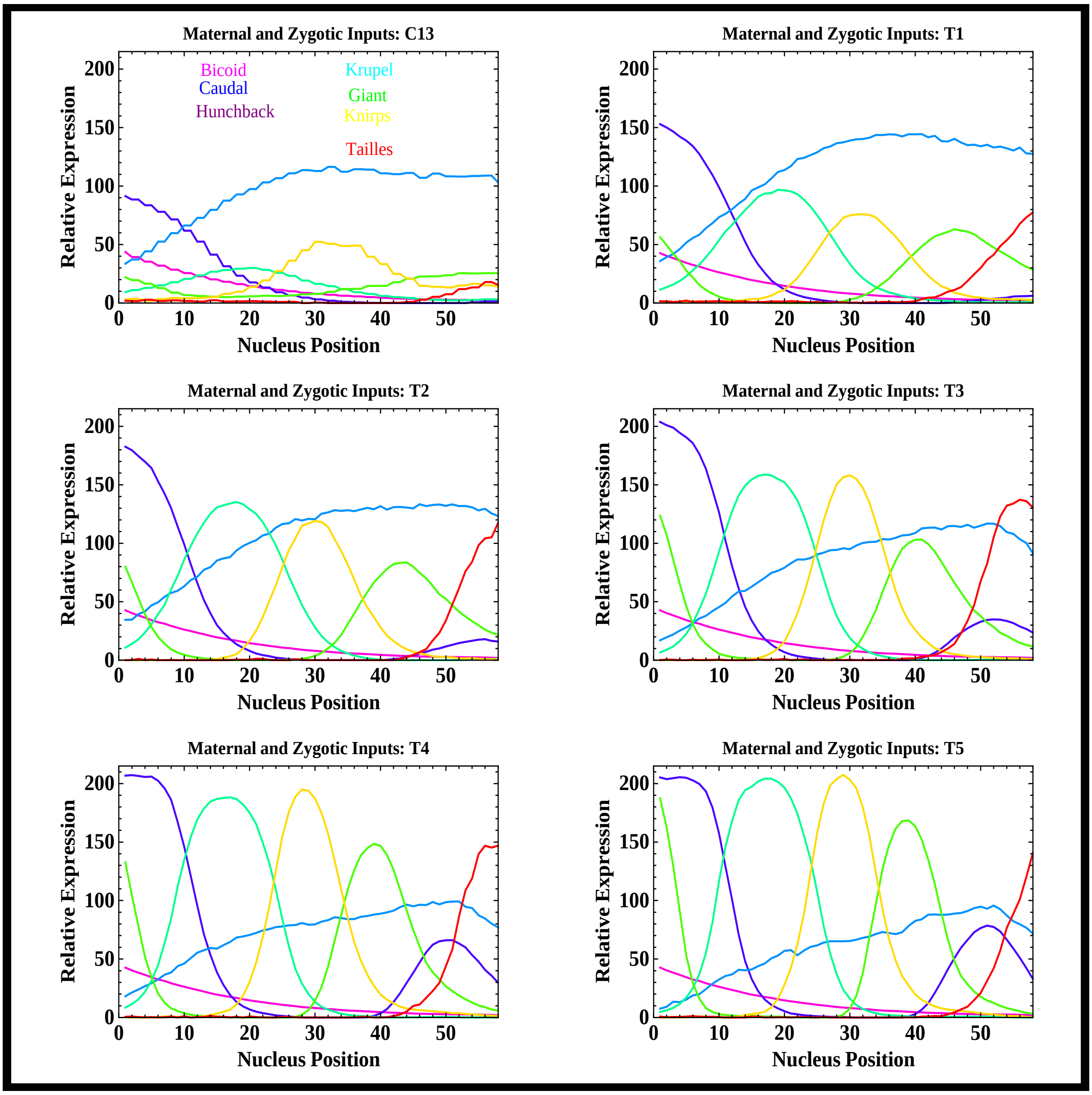

Supplement: Figure S1 — GAP Network: Wild Type Spatio-Temporal Expression Trajectories. Snapshots of wild type expression domains of the GAP network at different time points during early Drosophila embryogenesis. This network encompasses transcriptional regulators of maternal and zygotic origin, such as Bicoid, Caudal, Hunchback, Krupel, Giant, Knirps, Tailles, which get involved in complex patterns of cross-regulation, and subsequently provide regulatory inputs to downstream expression cascades of the segmentation network. C13 to T5 indicate time points along the expression trajectory of the network, during early Drosophila embryogenesis. This time window covers early to late stages of cleavage cycle (nuclear division) 14A of the Drosophila blastoderm. C13 = 40.250 mins; T1 = 53.925 mins; T2 = 60.175 mins; T3 = 66.425 mins; T4 = 72.675 mins; T5 = 78.925 mins. Expression Data from [29], [37] (0.03 MB PDF) [file pone.0006823.s001.pdf]
