# Supplementary material for: A Developmental Systems Perspective on Epistasis: Computational Exploration of Mutational Interactions in Model Developmental Regulatory Networks
Source: PLoS One. 2009 Sep 7;4(9):e6823. doi: 10.1371/journal.pone.0006823 (PMC2734181; doi:10.1371/journal.pone.0006823)
Supplement: Figures S2 — Representative Spatio-Temporal Expression Trajactories: Functional Networks. (0.30 MB PDF) [file pone.0006823.s002.pdf]

A Developmental Systems Perspective on Epistasis: Computational Exploration of Mutational Interactions in Model Developmental Regulatory Networks

Jayson Gutiérrez

Supporting Information Figure S2.

Representative Spatio-Temporal Expression Trajectories: Functional Networks

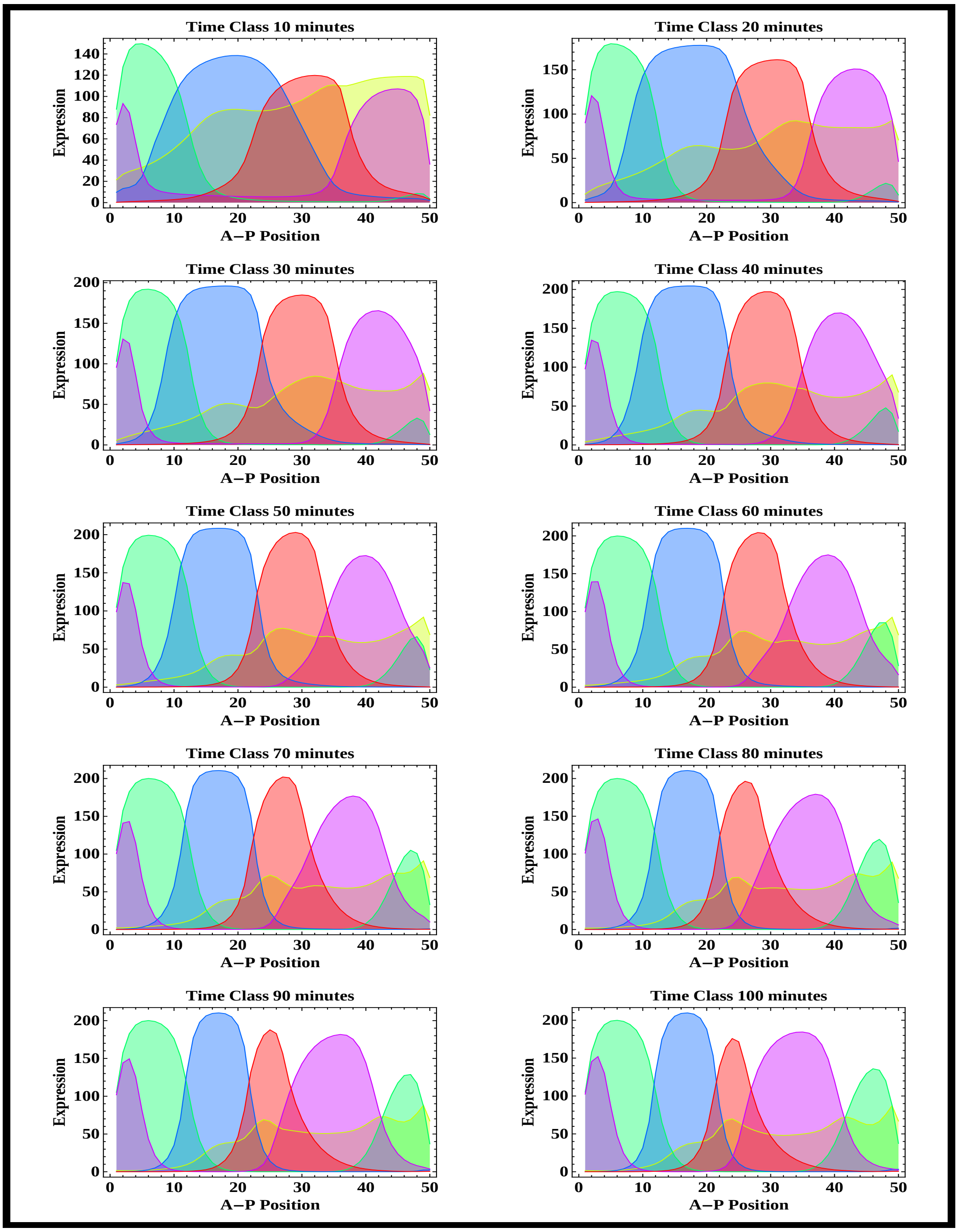

Figure S2A: Snapshots of expression domains at different time points during the virtual developmental time window of 100 minutes simulated in this study. Spatio-temporal expression dynamics for a functional network encompassing 5 transcriptional regulators

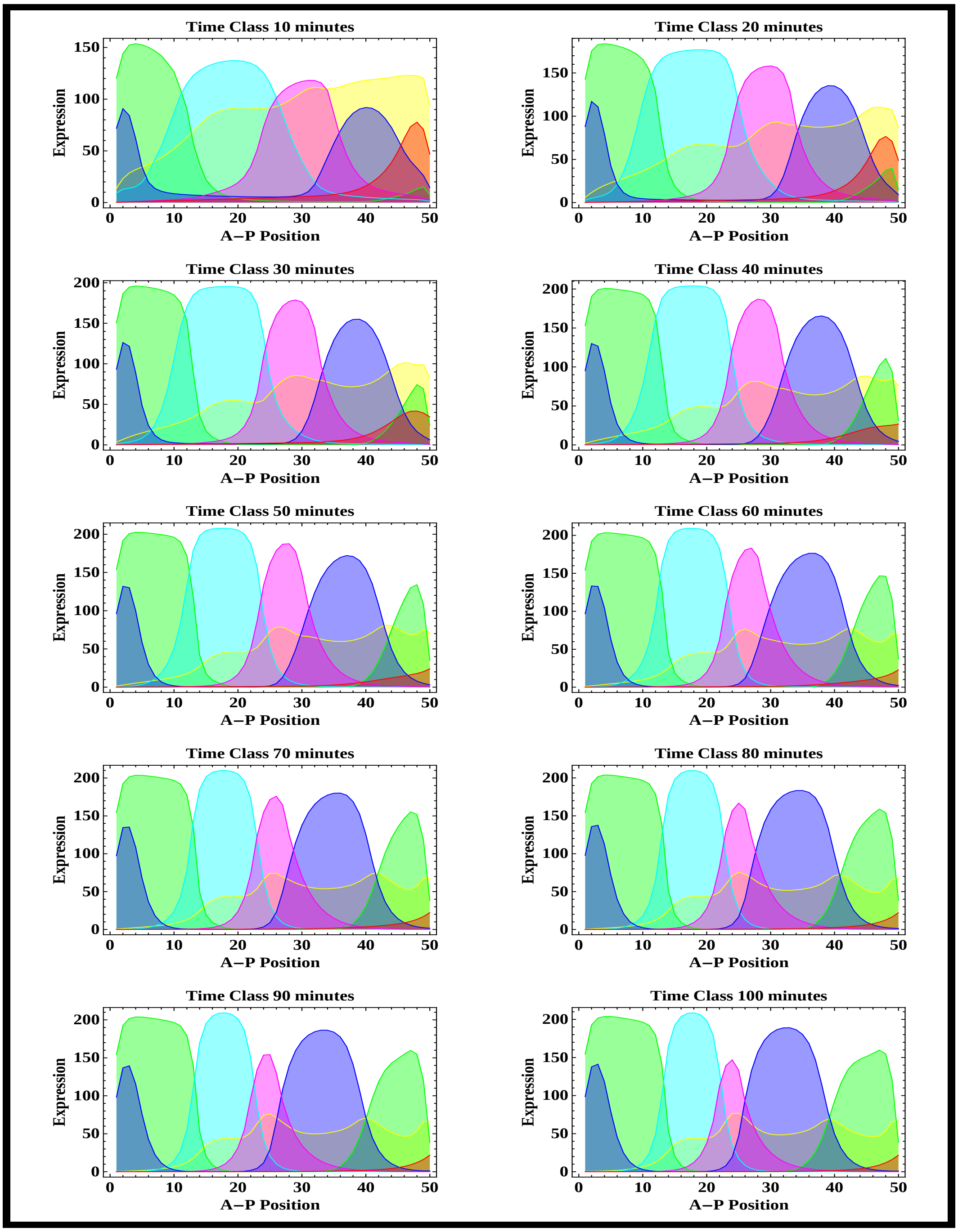

Figure S2B: Snapshots of expression domains at different time points during the virtual developmental time window of 100 minutes simulated in this study. Spatio-temporal expression dynamics for a functional network encompassing 6 transcriptional regulators

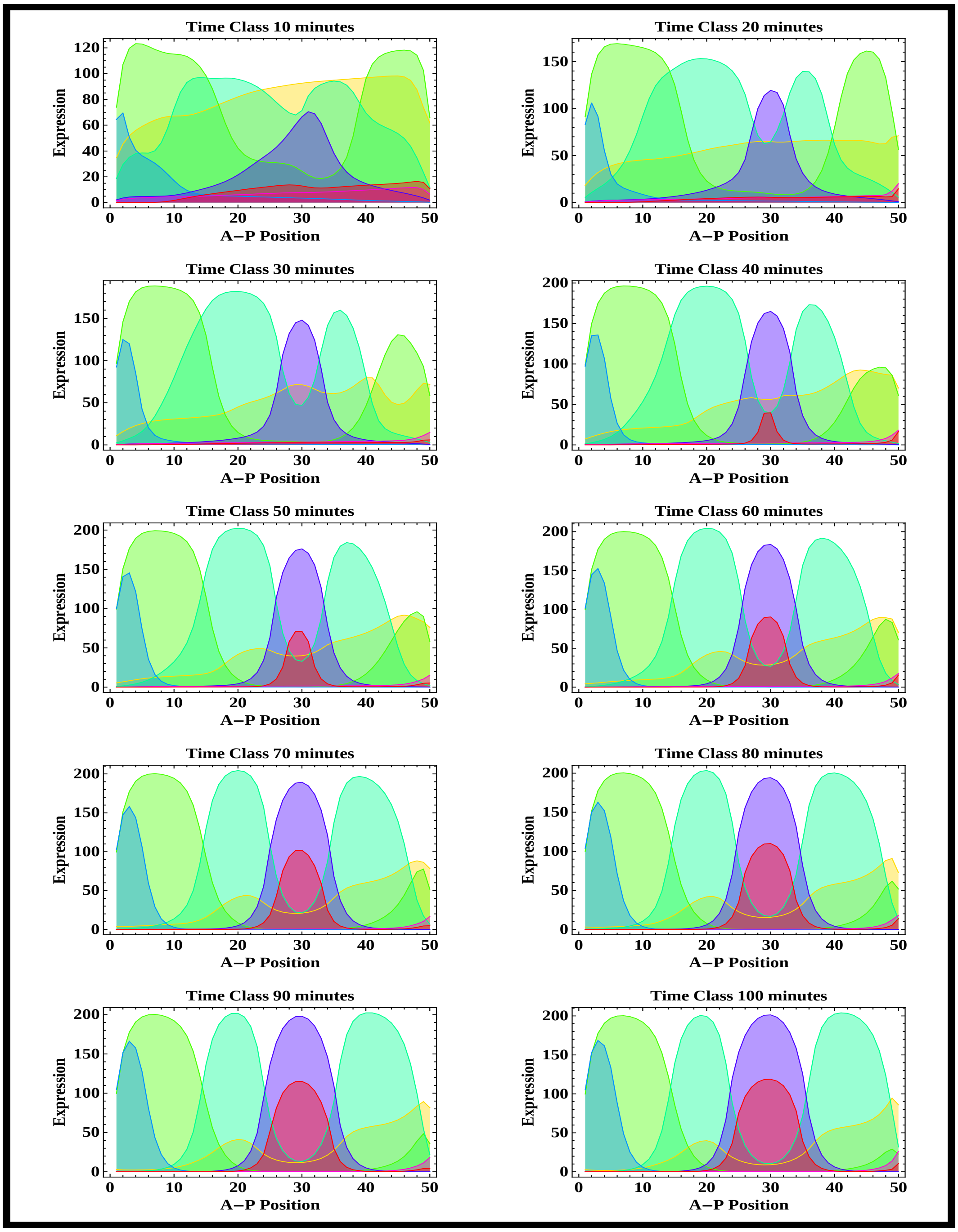

Figure S2C: Snapshots of expression domains at different time points during the virtual developmental time window of 100 minutes simulated in this study. Spatio-temporal expression dynamics for a functional network encompassing 7 transcriptional regulators

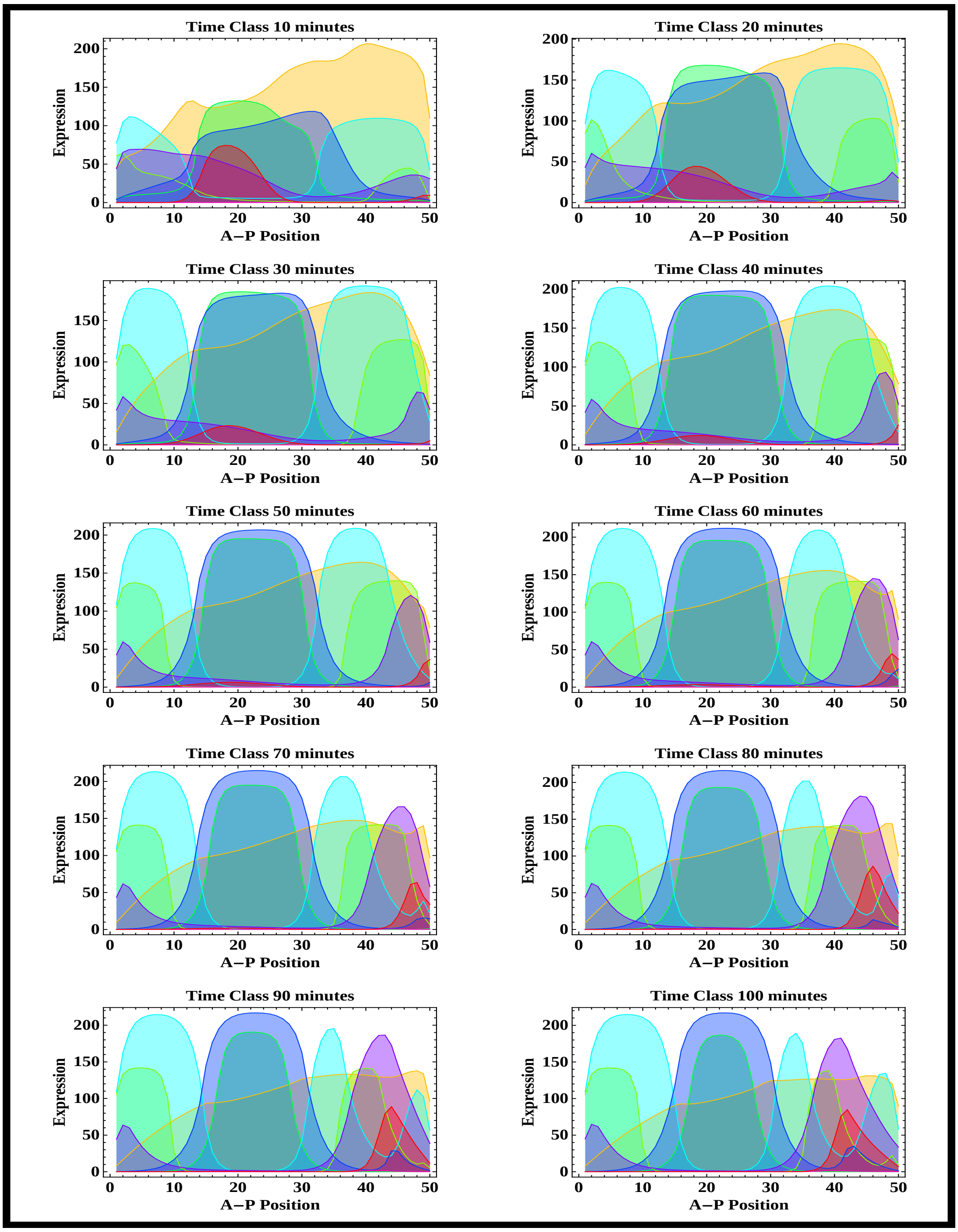

Figure S2D: Snapshots of expression domains at different time points during the virtual developmental time window of 100 minutes simulated in this study. Spatio-temporal expression dynamics for a functional network encompassing 8 transcriptional regulators
