# Supplementary material for: A Developmental Systems Perspective on Epistasis: Computational Exploration of Mutational Interactions in Model Developmental Regulatory Networks
Source: PLoS One. 2009 Sep 7;4(9):e6823. doi: 10.1371/journal.pone.0006823 (PMC2734181; doi:10.1371/journal.pone.0006823)
Supplement: Figures S3 — Representative Spatio-Temporal Expression Trajactories: Arbitrary Networks. (0.29 MB PDF) [file pone.0006823.s003.pdf]

A Developmental Systems Perspective on Epistasis: Computational Exploration of Mutational Interactions in Model Developmental Regulatory Networks

Jayson Gutiérrez

Supporting Information Figure S3.

Representative Spatio-Temporal Expression Trajectories: Arbitrary Networks

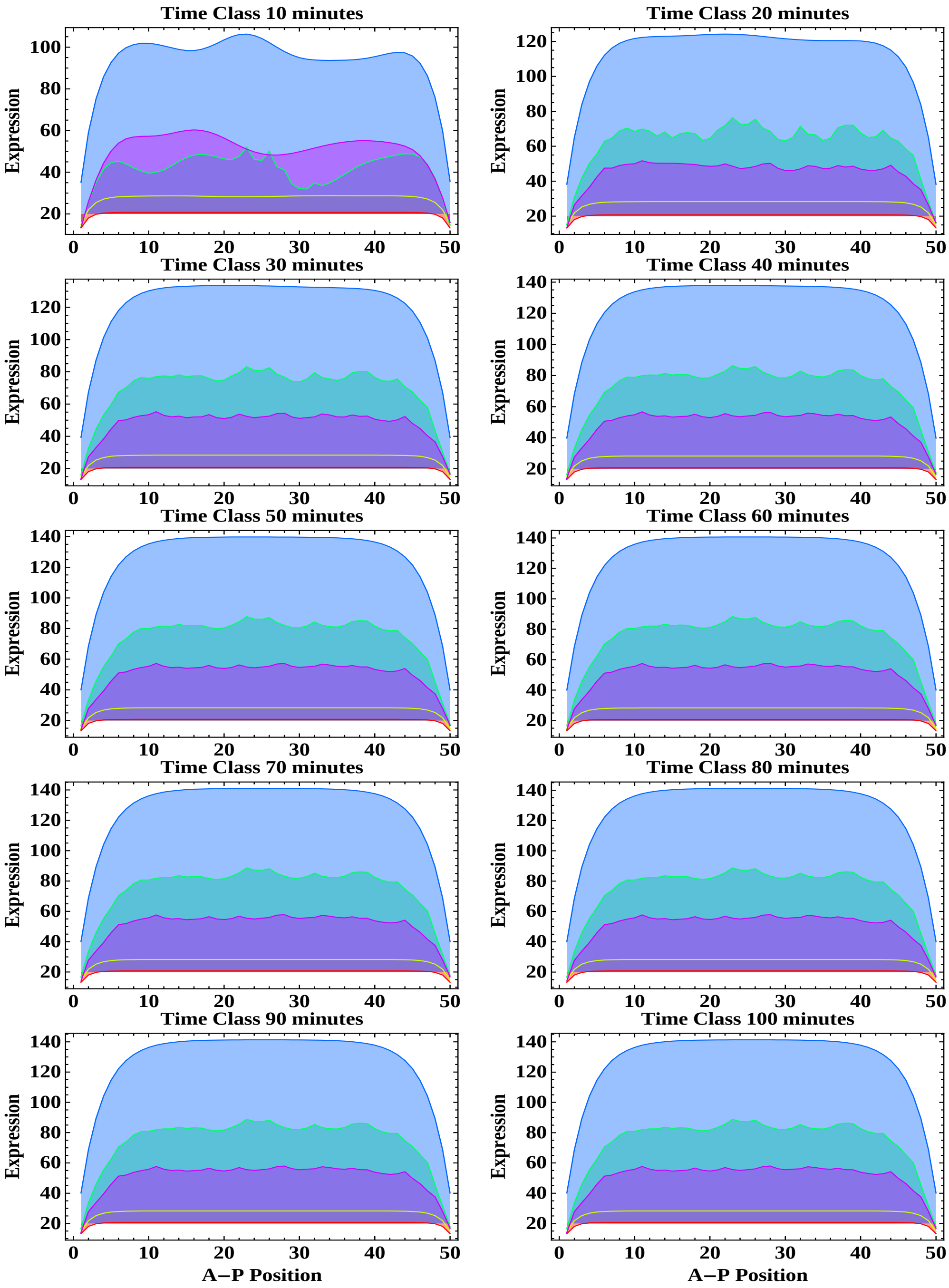

Figure S3A: Snapshots of expression domains at different time points during the virtual developmental time window of 100 minutes simulated in this study. Spatio-temporal expression dynamics for an arbitrary network encompassing 5 transcriptional regulators

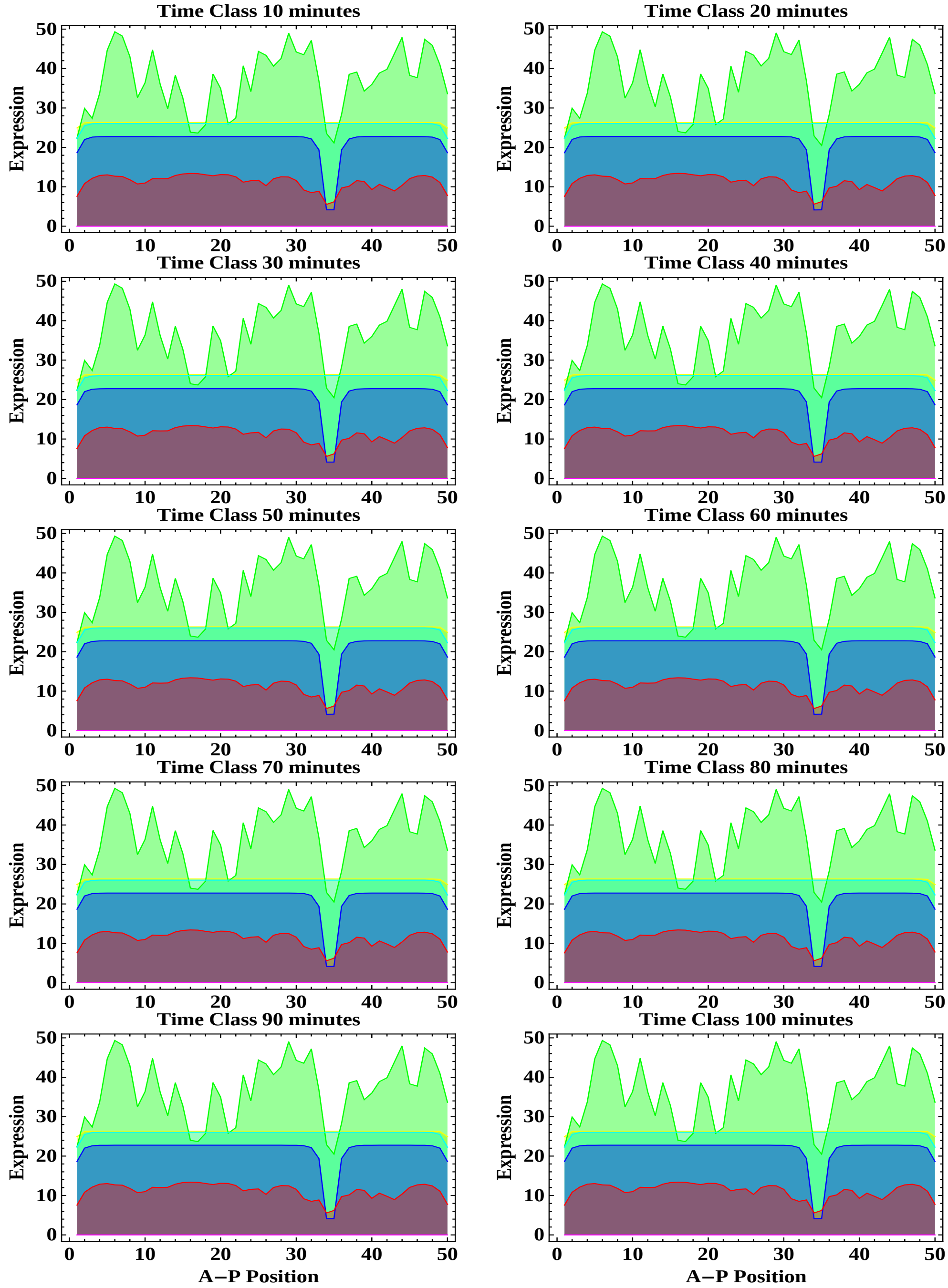

Figure S3B: Snapshots of expression domains at different time points during the virtual developmental time window of 100 minutes simulated in this study. Spatio-temporal expression dynamics for an arbitrary network encompassing 6 transcriptional regulators

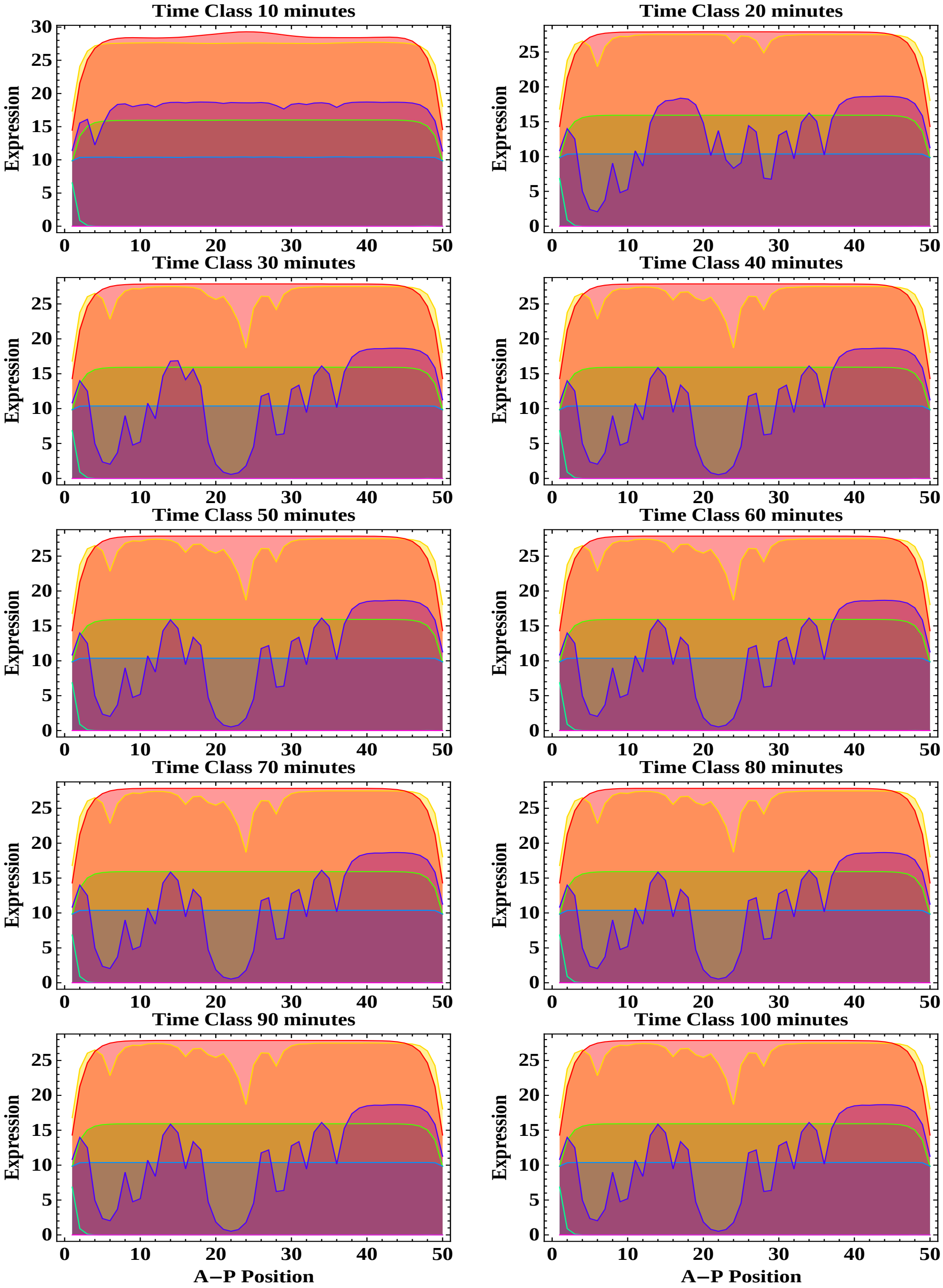

Figure S3C: Snapshots of expression domains at different time points during the virtual developmental time window of 100 minutes simulated in this study. Spatio-temporal expression dynamics for an arbitrary network encompassing 7 transcriptional regulators

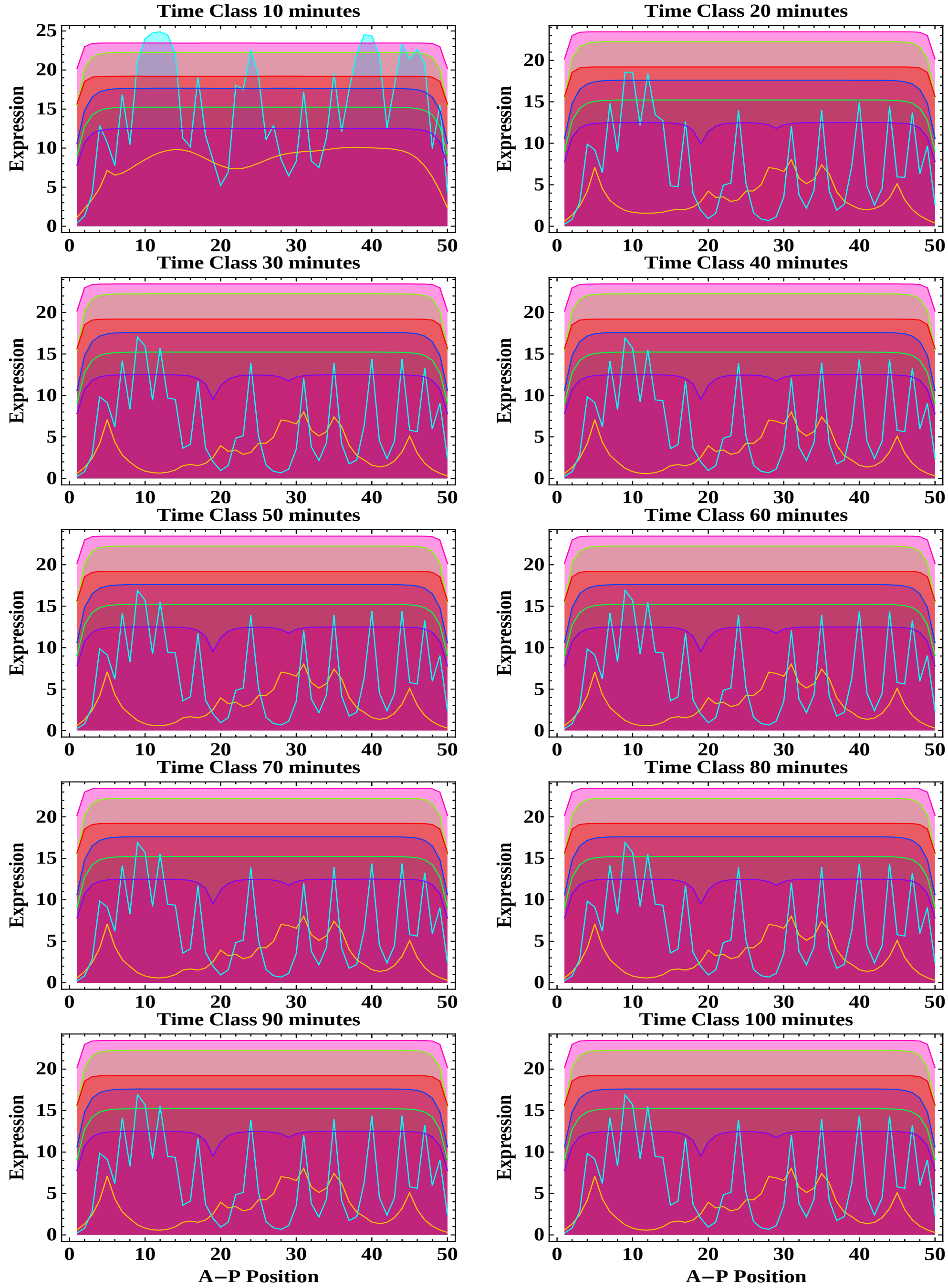

Figure S3D: Snapshots of expression domains at different time points during the virtual developmental time window of 100 minutes simulated in this study. Spatio-temporal expression dynamics for an arbitrary network encompassing 8 transcriptional regulators
