# Supplementary material for: A Developmental Systems Perspective on Epistasis: Computational Exploration of Mutational Interactions in Model Developmental Regulatory Networks
Source: PLoS One. 2009 Sep 7;4(9):e6823. doi: 10.1371/journal.pone.0006823 (PMC2734181; doi:10.1371/journal.pone.0006823)
Supplement: Figures S4 — Exemplar Network Topologies: Functional Networks. (0.19 MB PDF) [file pone.0006823.s004.pdf]

A Developmental Systems Perspective on Epistasis: Computational Exploration of Mutational Interactions in Model Developmental Regulatory Networks

Jayson Gutiérrez

Supporting Information Figure S4.

Exemplar Network Topologies: Functional Networks

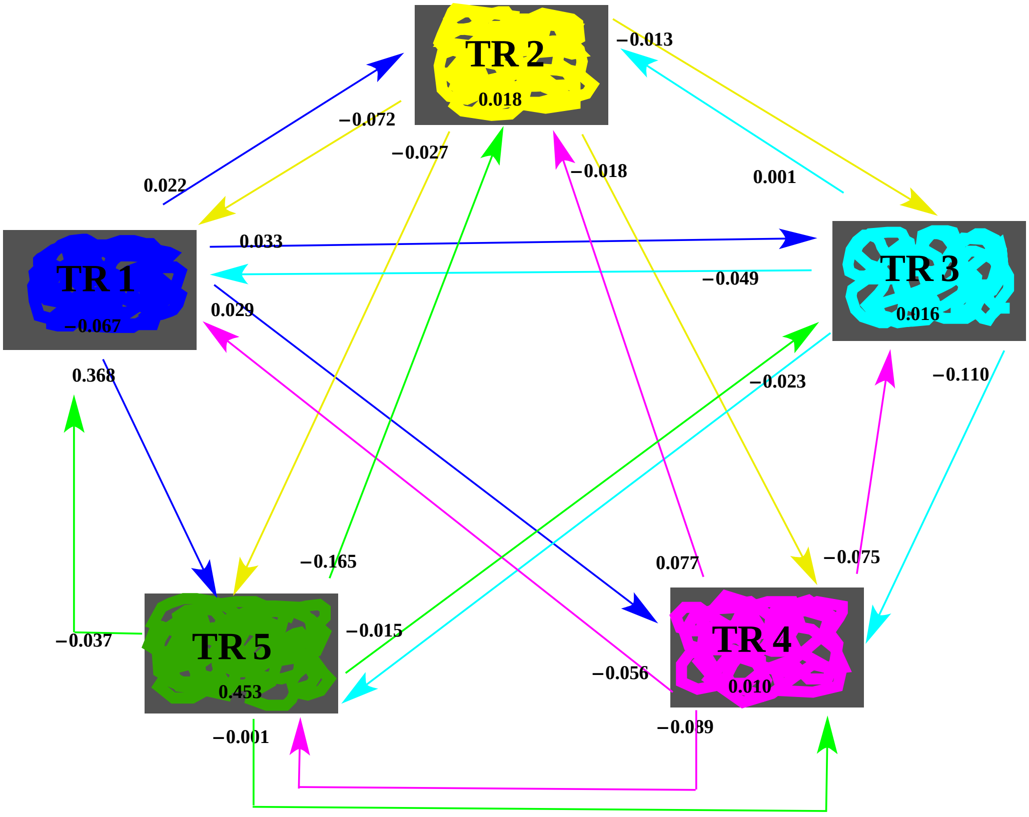

Figure S4A: This fully-interconnected network defines a functional topology encompassing 5 transcriptional regulators, and 25 regulatory interactions in total. Numbers indicate quantitative interactions among transcriptional regulators (TRs). Under this network configuration 10 different basic feedback motifs are realizable.

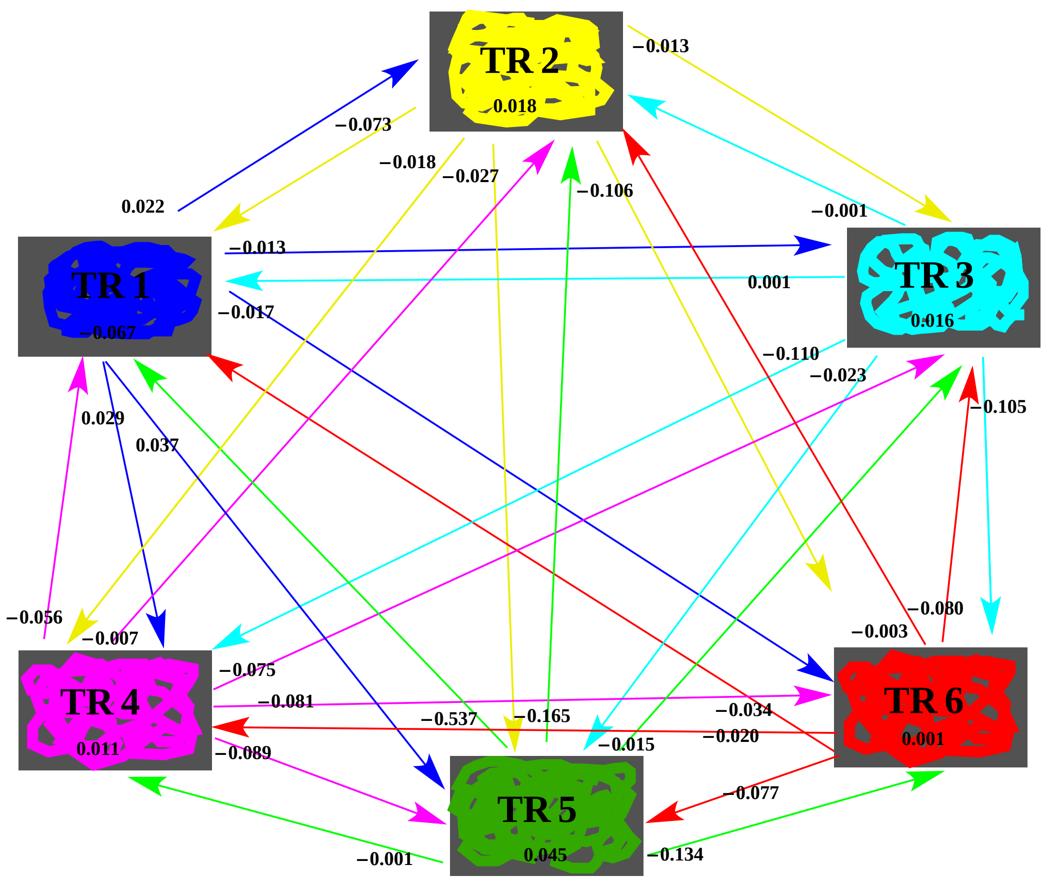

Figure S4B: This fully-interconnected network defines a functional topology encompassing 6 transcriptional regulators, and 36 regulatory interactions in total. Under this network configuration 15 different basic feedback motifs are realizable.

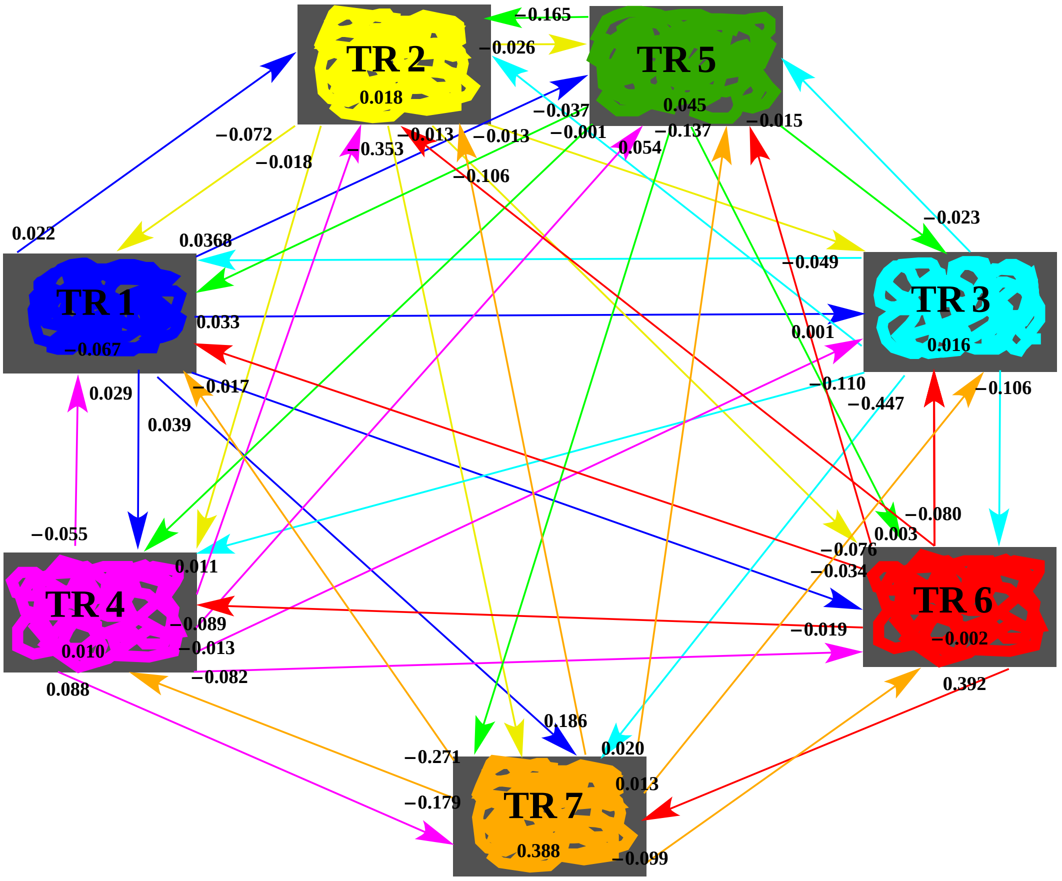

Figure S4C: This fully-interconnected network defines a functional topology encompassing 7 transcriptional regulators, and 49 regulatory interactions in total. Under this network configuration 21 different basic feedback motifs are realizable.

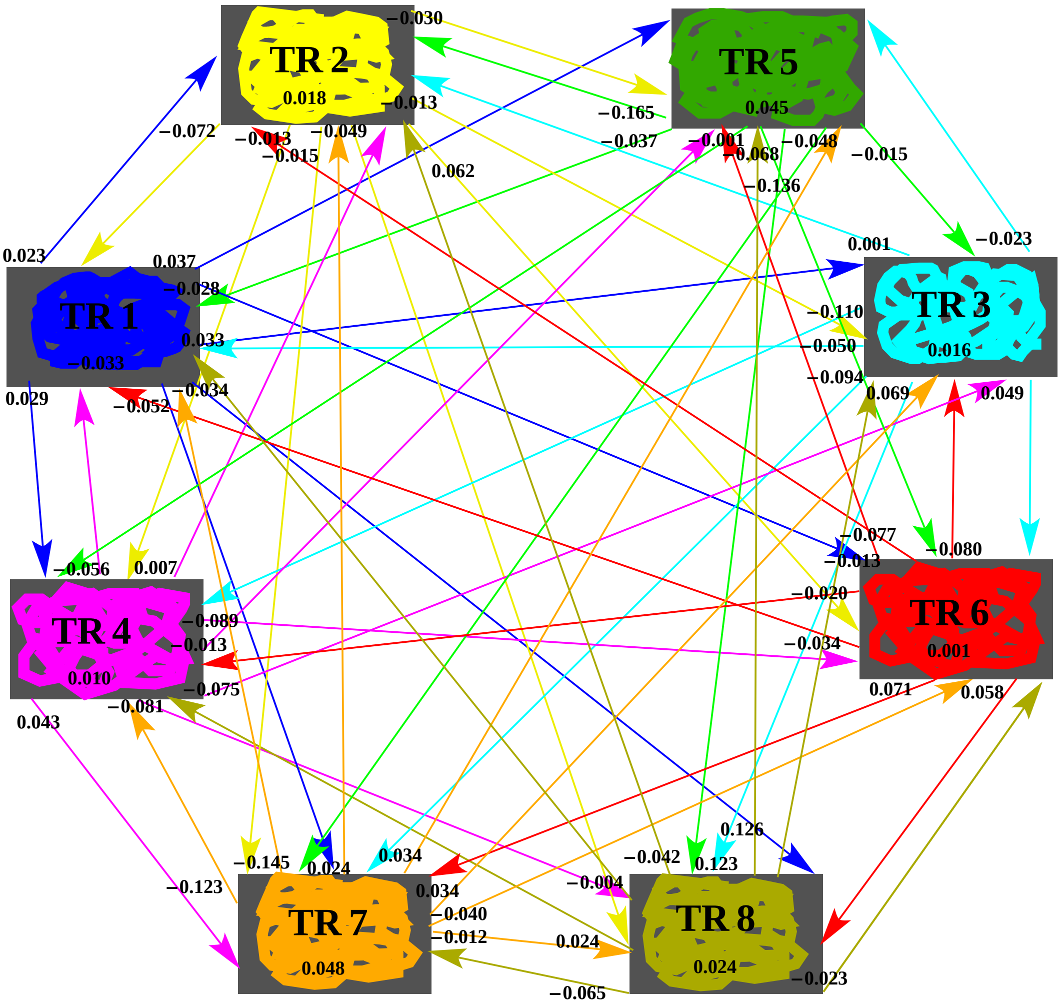

Figure S4D: This fully-interconnected network defines a functional topology encompassing 8 transcriptional regulators, and 64 regulatory interactions in total. Under this network configuration 28 different basic feedback motifs are realizable.
