# Supplementary material for: A Developmental Systems Perspective on Epistasis: Computational Exploration of Mutational Interactions in Model Developmental Regulatory Networks
Source: PLoS One. 2009 Sep 7;4(9):e6823. doi: 10.1371/journal.pone.0006823 (PMC2734181; doi:10.1371/journal.pone.0006823)
Supplement: Method S1 — (0.03 MB PDF) [file pone.0006823.s005.pdf]

A Developmental Systems Perspective on Epistasis: Computational Exploration of Mutational Interactions in Model Developmental Regulatory Networks

Jayson Gutiérrez

Supporting Information: Method S1

Sampling of Functional and Arbitrary Network Configurations:

The space of network configurations (topologies) encompassing 5, 6, 7 and 8 TRs was randomly explored. This experiment was performed in an attempt to sampling both functional and arbitrary network configurations. In the case of functional networks, besides screening for the space of topologies, parameter spaces were also sampled (with configurations ranging between 40-88 parameters). Additonally, both initial expression states (pre-patterns) and maternal inputs were required to conform spatial gradients along the one-dimensional syncytium modeled (50 nuclei). In order to have some reference points to conduct the search space, I made use of experimental and simulation data generated in previous studies (see [1,2] and Tables S1 and S2). Initially, a large ensemble of networks from random searches was constructed, amounting to 10^6 network configurations. The patterning capabilitites of this ensemble of regulatory networks were analyzed both systematically and manually; a wide spectrum of expression dynamics was found, ranging from complex overlapping and alternating expression domains, similar to those exhibited by the GAP network, to spike-like and uniformly distributed spatio-temporal expression trajectories (arbitrary networks). Later, it was constructed sub-ensembles of 15 networks for each network configuration class of 5, 6, 7 and 8 TRs. Networks belonging to these sub-ensembles were chosen according to their spatio-temporal trajectories. Thus, 4 sub-ensembles (referred to as ensembles in the main text) were constructed, each with 15 functional networks selected according to their capabilitites of accomplishing the patterning tasks of the GAP network. On the other hand, arbitrary network configurations were sampled randomly with the data considered in the selection process of the functional networks. Initially, an ensemble of 1000 arbitrary network configurations was constructed. Subsequently, I analyzed manually their expression dynamics so as to make sure that they did not meet the patterning requirements imposed for the functional networks. Similarly, I randomly chose 4 sub-ensembles of 15 networks for each network configuration class of 5, 6, 7 and 8 TRs as I did with functional configurations, for mutational analysis purposes.

1 References

1. Jaeger J, *et al.,* (2004) Dynamical Analysis of Regulatory Interactions in the GAP Gene System of *Drosophila melanogaster*. Genetics 167: 1721-1737.

2. Jaeger J, *et al.,* (2004) Dynamic Control of Positional Information in the Early *Drosophila* Embryo. Nature 430: 368-371.
