# Supplementary material for: A Developmental Systems Perspective on Epistasis: Computational Exploration of Mutational Interactions in Model Developmental Regulatory Networks
Source: PLoS One. 2009 Sep 7;4(9):e6823. doi: 10.1371/journal.pone.0006823 (PMC2734181; doi:10.1371/journal.pone.0006823)
Supplement: Method S2 — (0.04 MB PDF) [file pone.0006823.s006.pdf]

A Developmental Systems Perspective on Epistasis: Computational Exploration of Mutational Interactions in Model Developmental Regulatory Networks

Jayson Gutiérrez

Supporting Information: Method S2

Vicinity in Parameter Space and Evaluation of Mutational Interaction Patterns: The vicinity of both operating and reference points in parameter space were explored by systematically inducing perturbations in the regulatory interaction matrices of the network models considered. An operating point in parameter space can be seen as a parameter configuration of any network categorized as functional, whereas a reference point is defined by a parameter configuration corresponding to an arbitrary network. Perturbations in the regulatory interaction matrices were induced as follows: a randomly chosen matrix element W^{ab} was transformed into another functional element in this way $W^{ab} \rightarrow W^{ab} * \Theta$, with Θ being a mutational factor randomly sampled from the uniform distribution (0,2]. Given that the statistical distribution of potential mutational effects altering parameter values in regulatory networks is uncertain, I thus opted for sampling the space uniformly. It was evaluated the effects that multiple perturbations in the regulatory interaction matrix of the networks excerted on the reproducibility of an assumed *optimal* spatio-temporal expression trajectory (reference phenotype), generated by either a functional or an arbitrary network. Via an objective function, *phenotypic discrepancies* between a reference network and a mutant version of it were assessed (see below). Previous rounds of *in silico* analysis of the ensemble of networks showed that phenotypic discrepancies below a threshold value (phenotypic discrepancies ≤ 1) were indicative of irrelevant mutational effects with respect to the reproducibility of the expression trajectories (data not shown). To put it in a developmental context, this means that a single, or combined effect of multiple perturbations in the regulatory networks resulted in minimal patterning deffects. Subsequently, fitness values were evaluated via a transfer function (see below) that assigns a value ranging in [0,1] to each mutant network, according to its phenotypic discrepancy value. To summarize, the single, or combined effect of multiple perturbations on the reproducibility of an assumed "optimal" spatio-temporal expression trajectory displayed by a functional or an arbitrary network, was evaluated at the fitness level. Mutational interactions were thus evaluated according to these fitness values (fitness epistasis) via two standard methods widely used in the literature (see [1,2]). It is worth mentioning that such fitness evaluations relied on the assumption that any mutational perturbation simulated in the regulatory networks might lead to one of two possible situations: 1) recovering of the maximum attainable fitness value of 1 after the introduction of previous mutations, or 2) harmful effects, whereby mutations tend to induce decreases in fitness, consistently. Thus, in my modeling framework I did not account for mutations whose effects might improve a fitness value above 1. Finally, fitness effects of mutational combinations were evaluated under the assumption

of constant environmental conditions (see Method S3).

1 References

1. Lenski RE, Ofria C, Collier TC, Adami C, (1999) Genome Complexity, Robustness and Genetic Interactions in Digital Organisms. Nature 400: 661-664.

2. Mani R, St. Onge RP, Hartman JL, Giaever G, Roth FP, (see 2008) Defining Genetic Interaction. PNAS 105: 3461-3466.
