# Supplementary material for: A Developmental Systems Perspective on Epistasis: Computational Exploration of Mutational Interactions in Model Developmental Regulatory Networks
Source: PLoS One. 2009 Sep 7;4(9):e6823. doi: 10.1371/journal.pone.0006823 (PMC2734181; doi:10.1371/journal.pone.0006823)
Supplement: Method S3 — (0.05 MB PDF) [file pone.0006823.s007.pdf]

A Developmental Systems Perspective on Epistasis: Computational Exploration of Mutational Interactions in Model Developmental Regulatory Networks

Jayson Gutiérrez

Supporting Information: Method S3

Evaluation of Phenotypic Discrepancies and Fitness: The phenotypic consequences of multiple and single perturbations were evaluated as discrepancies between the mutant ($\mathbf{Y}_{Mut}(t)$) and the corresponding optimal ($\mathbf{Y}_{Opt}(t)$), or reference ($\mathbf{Y}_{Ref}(t)$), expression trajectory displayed by a functional, or an arbitrary network, respectively. Such discrepancies were assessed via a multidimensional dynamic Euclidean metric of this form :

$$PD(\mathbf{Y}_{Opt}(t), \mathbf{Y}_{Mut}(t)) = \frac{\sum_{t=1}^{100} \sum_{a=1}^{N} \sqrt{\sum_{i=1}^{50} (y_{i,Opt}^{a}(t) - y_{i,Mut}^{a}(t))^2}}{100 * N * 50} \quad (1)$$

Where $PD(\mathbf{Y}_{Opt}(t), \mathbf{Y}_{Mut}(t))$ stands for the phenotypic discrepancy between a single or multiple mutant network and the corresponding unperturbed functional network (or arbitrary network $PD(\mathbf{Y}_{Ref}(t), \mathbf{Y}_{Mut}(t))$). N gives the number of TRs of a network model. Note that this dynamic metric allows for the evaluation of changes in the concentration of each transcriptional regulator within each nucleus modeled, every minute during the virtual time window simulated (100 minutes). Thus, the phenotypic effects of single or multiple mutations were explored via this dynamic metric, at high temporal and spatial resolution in a virtual embryo (a strip of 50 nuclei). In turn, this metric provided the arguments for a function aimed for evaluating the fitness of a virtual embryo carrying a mutant regulatory network, as follows:

$$F(\mathbf{Y}_{Mut}(t)) = \begin{cases} 1 & \text{iff} \quad PD(\mathbf{Y}_{Opt}(t), \mathbf{Y}_{Mut}(t)) \leq 1 \\ exp(-PD(\mathbf{Y}_{Opt}(t), \mathbf{Y}_{Mut}(t))) & \text{otherwise} \end{cases} \quad (2)$$

This fitness function was built on the basis of experimental observations in the *Drosophila* embryo, which can be thought of as a naive approximation intended to connect patterning deffects and the viability of a virtual mutant embryo. For instance, experimental observations suggest that absolute concentrations of GAP proteins are thought to be no critical for the viability of an embryo, since reducing the concentration of a GAP protein by a factor of 2 in a heterozygote induces slight changes in the relative position of an expression domain and its boundaries [1,2]. In other words, the segmentation

network seems to be capable of displaying buffering mechanisms, endowing the embryo with the capacity of compensating for a variety of perturbations, including variation in gene dosage due to hypomorphic mutations, and environmental fluctuations. Furthermore, previous computational analyses (personal observations, data not shown) indicated that $PD(\mathbf{Y}_{Opt}(t), \mathbf{Y}_{Mut}(t)) \leq 1$ corresponded, approximately, to a 10 percent of discrepancies in expression patterns. Perturbed expression patterns falling below this cut-off were considered neutral mutants, since they exhibited negligible patterning defects, such as sligth expansion of expression domains (between 1-2 nuclei), slight increments in the concentration of TRs within one or several spatial expression domains, and small time delays (heterochronies) in the expression of one or more TRs during the time window simulated. It is important to note that these patterning errors have been found to be natural components of phenotypic variation in populations of embryos with apparently equal probabilities of survival [3]. Thus, it was assumed that above this threshold of phenotypic discrepancy ($PD(\mathbf{Y}_{Opt}(t), \mathbf{Y}_{Mut}(t)) > 1$) considerable patterning errors with very likely adaptive costs (i.e. embryonic lethality) under common and extreme mutational conditions, emerged. These ideas are indeed reasonable in terms of fitness costs, as an early patterning network such as the GAP system convey, most of the time, considerably noisy positional information that is filtered and read-out by downstrean regulatory layers (pair-rule and segment polarity networks), which are essential for the unfolding of the final stage of segment patterning. Nevertheless, possible relationships between developmental patterning errors and their fitness costs remain puzzling, and represent a major concern in evolutionary developmental biology and evolutionary systems biology studies.

1 References

1. Nusslein-Volhard, Wieschaus E, (1980) Mutations affecting segment number and polarity in *Drosophila.* Nature 287: 795-801.

2. Reinitz J, Mjolsness E, Sharp DH, (1995) Model for Cooperative Control of Positional Information in *Drosophila* by Bicoid and Maternal Hunchback. Experimental Zoology 271: 47-56.

3. Surkova S, *et al.,* (2008) Characterization of the *Drosophila* Segment Determination Morphome. Developmental biology 313: 844-862.
