# Supplementary material for: A Developmental Systems Perspective on Epistasis: Computational Exploration of Mutational Interactions in Model Developmental Regulatory Networks
Source: PLoS One. 2009 Sep 7;4(9):e6823. doi: 10.1371/journal.pone.0006823 (PMC2734181; doi:10.1371/journal.pone.0006823)
Supplement: Method S4 — (0.02 MB PDF) [file pone.0006823.s008.pdf]

A Developmental Systems Perspective on Epistasis: Computational Exploration of Mutational Interactions in Model Developmental Regulatory Networks

Jayson Gutiérrez

Supporting Information: Method S4

Simulated Temporal Scales and Dynamical Regimes of Regulatory Networks: Expression trajectories were simulated during a predefined virtual time window of 100 minutes (a developmentally-relevant time scale similar to that reported for the wild-type GAP network). According to this temporal scale, the dynamics of the functional networks were required to enter into quasi-stationary expression states, characterized by the confinement of expression domains and their boundaries within certain regions along the one-dimensional syncytium modeled, after minute 70 of virtual developmental time. Thus, functional network configurations were characterized by the capability of generating transient spatio-temporal expression patterns during the first minutes simulated, and quasi-stationary dynamics after minute 70 (see figures S2A-S2D). This was done in an attempt to be as rigorous as possible with the assessment of mutational effects at both the phenotypic and fitness level. This is a more biologically realistic alternative than common steady state analysis, essentially, for two reasons: 1) The phase space of these multidimensional developmental systems tend to be quite complex, in which strange topological structures (i.e. dynamical attractors) emerge naturally, thus precluding any possibility of analytic characterization. 2) The dynamic nature of early developmental trajectories is characterized by transient expression dynamics, rather than asymptotically dynamic regimes. Finally, arbitrary network configurations were imposed to exhibit quasi-stationary dynamics at most after minute 20 of virtual developmental time (see figures S3A-S3D).

Models and algorithms were coded in Mathematica 6.0 (Wolfram Research), and run in 4 work stations with intel core 2 Duo processors, under the Unix environment.
