# Supplementary material for: A Developmental Systems Perspective on Epistasis: Computational Exploration of Mutational Interactions in Model Developmental Regulatory Networks
Source: PLoS One. 2009 Sep 7;4(9):e6823. doi: 10.1371/journal.pone.0006823 (PMC2734181; doi:10.1371/journal.pone.0006823)
Supplement: Tables S1 — (0.06 MB PDF) [file pone.0006823.s009.pdf]

A Developmental Systems Perspective on Epistasis: Computational Exploration of Mutational Interactions in Model Developmental Regulatory Networks

Jayson Gutiérrez

Supporting Information: Table S1

Reference regulatory (W^{ab}) parameter values around which random searches through parameter space were conducted. Reference from [1,2]. Regulatory interactions among the transcription factors encoding genes of the GAP system: *caudal* (cad), *hunchback* (hb), *Kruppel* (Kr), *giant* (gt), *knirps* (kni), and *tailles* (tll).

Value / W^{ij}	Reference 1	Reference 2	Reference 3	Reference 4	Reference 5
$W^{cad \leftarrow cad}$	−0.058	−0.061	−0.078	−0.068	−0.161
$W^{cad \leftarrow hb}$	−0.064	−0.071	−0.097	−0.073	−0.134
$W^{cad \leftarrow Kr}$	−0.045	−0.042	−0.059	−0.050	−0.082
$W^{cad \leftarrow gt}$	−0.054	−0.044	−0.062	−0.056	−0.078
$W^{cad \leftarrow kni}$	−0.035	−0.020	−0.045	−0.038	−0.066
$W^{cad \leftarrow tll}$	−0.033	−0.016	−0.030	−0.034	−0.030
$W^{hb \leftarrow cad}$	0.024	0.026	0.029	0.022	0.035
$W^{hb \leftarrow hb}$	0.017	0.022	0.020	0.019	0.013
$W^{hb \leftarrow Kr}$	0.001	0.002	0.003	0.000	0.002
$W^{hb \leftarrow gt}$	0.012	0.006	0.004	0.011	−0.003
$W^{hb \leftarrow kni}$	−0.189	−0.141	−0.193	−0.166	−0.141
$W^{hb \leftarrow tll}$	0.003	0.004	−0.001	0.003	−0.005
$W^{Kr \leftarrow cad}$	0.0034	0.042	0.024	0.033	0.037
$W^{Kr \leftarrow hb}$	−0.016	−0.002	−0.006	−0.014	−0.035
$W^{Kr \leftarrow Kr}$	0.021	0.014	0.016	0.017	0.062
$W^{Kr \leftarrow gt}$	−0.068	−0.067	−0.036	−0.076	−0.008
$W^{Kr \leftarrow kni}$	−0.020	−0.014	−0.002	−0.015	−0.073
$W^{Kr \leftarrow tll}$	−0.066	−0.077	−0.082	−0.080	−0.062
$W^{gt \leftarrow cad}$	0.031	0.061	0.021	0.029	0.032
$W^{gt \leftarrow hb}$	−0.020	0.010	−0.025	−0.018	−0.008
$W^{gt \leftarrow Kr}$	−0.120	−0.154	−0.112	−0.310	−0.152
$W^{gt \leftarrow gt}$	0.011	0.000	0.015	0.011	0.020
$W^{gt \leftarrow kni}$	−0.002	−0.003	0.004	−0.001	−0.002
$W^{gt \leftarrow tll}$	−0.021	−0.140	0.006	−0.020	−0.016
$W^{kni \leftarrow cad}$	0.040	0.053	0.030	0.037	0.053
$W^{kni \leftarrow hb}$	−0.020	−0.033	−0.015	−0.027	−0.011
$W^{kni \leftarrow Kr}$	−0.025	−0.037	−0.030	−0.021	−0.023
$W^{kni \leftarrow gt}$	−0.108	−0.160	−0.080	−0.090	−0.061
$W^{kni \leftarrow kni}$	0.043	0.046	0.054	−0.045	0.032
$W^{kni \leftarrow tll}$	−0.117	−0.011	−0.045	−0.077	−0.115
$W^{tll \leftarrow cad}$	0.003	0.099	−0.058	−0.018	0.173
$W^{tll \leftarrow hb}$	−0.062	−0.006	−0.133	−0.106	−0.005
$W^{tll \leftarrow Kr}$	−0.043	−0.176	−0.035	−0.106	−0.067
$W^{tll \leftarrow gt}$	−0.059	−0.010	−0.070	−0.082	−0.104
$W^{tll \leftarrow kni}$	−0.156	−0.145	−0.049	−0.137	−0.071
$W^{tll \leftarrow tll}$	−0.003	0.012	0.017	0.003	0.023

1 References

1. Jaeger J, *et al.,* (2004) Dynamical Analysis of Regulatory Interactions in the GAP Gene System of *Drosophila melanogaster.* Genetics 167: 1721-1737.

2. Jaeger J, *et al.,* (2004) Dynamic Control of Positional Information in the Early *Drosophila* Embryo. Nature 430: 368-371.
