# Supplementary material for: A Developmental Systems Perspective on Epistasis: Computational Exploration of Mutational Interactions in Model Developmental Regulatory Networks
Source: PLoS One. 2009 Sep 7;4(9):e6823. doi: 10.1371/journal.pone.0006823 (PMC2734181; doi:10.1371/journal.pone.0006823)
Supplement: Tables S2 — (0.06 MB PDF) [file pone.0006823.s010.pdf]

A Developmental Systems Perspective on Epistasis: Computational Exploration of Mutational Interactions in Model Developmental Regulatory Networks

Jayson Gutiérrez

Supporting Information: Table S2

Reference parameter values around which random searches through parameter space were conducted. Reference from [1,2]. Maximal protein synthesis rate (K^a), Diffusion rate (D^a), and half-life ($t^a_{1/2}$) which determines the intrinsic degradation rate of a protein (ρ^a)

Parameter \ Value	Reference 1	Reference 2	Reference 3	Reference 4	Reference 5
K^{cad}	45.000	20.000	25.000	19.450	15.000
K^{hb}	15.000	20.000	19.308	20.459	17.885
K^{Kr}	14.701	15.363	20.000	16.373	12.181
K^{gt}	15.000	16.926	15.987	15.789	25.000
K^{kni}	14.870	11.638	11.234	12.185	16.949
K^{tll}	15.000	17.202	12.412	11.906	11.008
D^{cad}	0.200	0.300	0.263	0.200	0.300
D^{hb}	0.160	0.200	0.200	0.063	0.262
D^{Kr}	0.200	0.171	0.200	0.300	0.161
D^{gt}	0.128	0.086	0.148	0.142	0.300
D^{kni}	0.200	0.200	0.300	0.300	0.200
D^{tll}	0.300	0.300	0.300	0.200	0.200
$t^{cad}_{1/2}$	18.000	18.000	18.000	18.000	18.000
$t^{hb}_{1/2}$	9.631	7.043	7.131	7.254	8.453
$t^{Kr}_{1/2}$	10.081	10.260	7.791	8.980	12.571
$t^{gt}_{1/2}$	9.449	16.655	8.221	9.577	5.141
$t^{kni}_{1/2}$	13.287	14.258	13.482	12.498	9.040
$t^{tll}_{1/2}$	13.770	18.000	9.535	16.842	13.615

1 References

1. Jaeger J, *et al.,* (2004) Dynamical Analysis of Regulatory Interactions in the GAP Gene System of *Drosophila melanogaster.* Genetics 167: 1721-1737.

2. Jaeger J, *et al.,* (2004) Dynamic Control of Positional Information in the Early *Drosophila* Embryo. Nature 430: 368-371.
